# Vitrification and Nanowarming of Kidneys

**DOI:** 10.1002/advs.202101691

**Published:** 2021-08-11

**Authors:** Anirudh Sharma, Joseph Sushil Rao, Zonghu Han, Lakshya Gangwar, Baterdene Namsrai, Zhe Gao, Hattie L. Ring, Elliott Magnuson, Michael Etheridge, Brian Wowk, Gregory M. Fahy, Michael Garwood, Erik B. Finger, John C. Bischof

**Affiliations:** ^1^ Department of Mechanical Engineering University of Minnesota Minneapolis MN 55455 USA; ^2^ Department of Surgery University of Minnesota Minneapolis MN 55455 USA; ^3^ Center for Magnetic Resonance Research Department of Radiology University of Minnesota Minneapolis MN 55455 USA; ^4^ 21st Century Medicine Inc Fontana CA 92336 USA; ^5^ Department of Biomedical Engineering University of Minnesota Minneapolis MN 55455 USA

**Keywords:** cryopreservation, iron oxide nanoparticles, kidney, perfusion, radiofrequency warming, vitrification

## Abstract

Vitrification can dramatically increase the storage of viable biomaterials in the cryogenic state for years. Unfortunately, vitrified systems ≥3 mL like large tissues and organs, cannot currently be rewarmed sufficiently rapidly or uniformly by convective approaches to avoid ice crystallization or cracking failures. A new volumetric rewarming technology entitled “nanowarming” addresses this problem by using radiofrequency excited iron oxide nanoparticles to rewarm vitrified systems rapidly and uniformly. Here, for the first time, successful recovery of a rat kidney from the vitrified state using nanowarming, is shown. First, kidneys are perfused via the renal artery with a cryoprotective cocktail (CPA) and silica‐coated iron oxide nanoparticles (sIONPs). After cooling at −40 °C min^−1^ in a controlled rate freezer, microcomputed tomography (µCT) imaging is used to verify the distribution of the sIONPs and the vitrified state of the kidneys. By applying a radiofrequency field to excite the distributed sIONPs, the vitrified kidneys are nanowarmed at a mean rate of 63.7 °C min^−1^. Experiments and modeling show the avoidance of both ice crystallization and cracking during these processes. Histology and confocal imaging show that nanowarmed kidneys are dramatically better than convective rewarming controls. This work suggests that kidney nanowarming holds tremendous promise for transplantation.

## Graphical abstract

Vitrification dramatically increases organ storage times in the cryogenic state beyond current storage limits. However, vitrified kidneys cannot currently be rewarmed sufficiently rapidly or uniformly to avoid ice crystallization or cracking failures. A new radiofrequency rewarming technology called “nanowarming” addresses this problem by exciting magnetic nanoparticles to rewarm vitrified kidneys rapidly and uniformly.

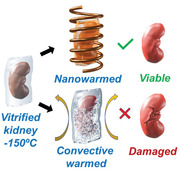

## 1 Introduction

The current clinical standard for organ preservation is cold storage on ice, which allows preservation for 24–36 h prior to transplant for kidneys.^[^ ^1^, ^2^, ^3^ ^]^ But this time window often results in primary graft dysfunction, shortened graft survival, and increased recipient mortality.^[^ ^1^ ^]^ Current efforts to extend safe preservation times include hypothermic machine perfusion,^[^ ^4^ ^]^ normothermic oxygenated perfusion,^[^ ^5^ ^]^ and supercooling;^[^ ^6^ ^]^ each of which may reduce preservation injury and prolong preservation times to 1–2 days. Tissue and organ cryopreservation at ultralow temperatures (≤−130 °C) has the potential to enable much longer term organ storage or true “organ banking,”^[^ ^1^, ^7^, ^8^ ^]^ which could significantly increase the number and quality of kidneys available for transplant and result in improved donor‐recipient matching.

**Figure 1:**
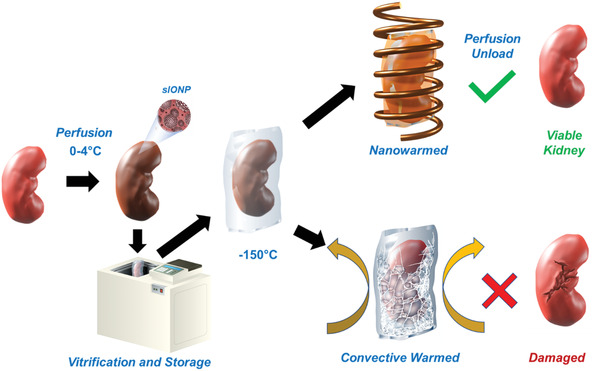
Schematic flow of kidney nanowarming. The kidney is hypothermically (0–4 °C) perfused with CPA (VS55) and sIONPs through the renal artery, then immersed in a cryobag containing VS55+sIONP and cooled rapidly to a vitrified state at −150 °C in a controlled rate freezer (CRF). During rewarming, convective warming in air or water‐bath will result in ice crystallization due to insufficient warming rates and/or cracking from thermomechanical gradients, thus damaging the kidney. In contrast, nanowarming of the kidney, using an RF magnetic field, results in rapid and uniform heating, minimizing cryopreservation damage that results in recovery similar to CPA load and unload only controls.

Currently, the chief barrier to cryopreserving a whole organ lies in warming, not cooling. Cryoprotective agents (CPAs) perfused through an organ, prevent damaging ice formation when they are cooled more rapidly than their critical cooling rates (CCRs, the rates necessary to outrun ice formation during cooling) or higher, which are now routinely achieved. However, the critical warming rates (CWRs) of such CPAs are orders of magnitude higher than their CCRs,^[^ ^9^ ^]^ and warming slower than the CWR dramatically increases the risk of lethal ice formation and irreversible damage to the organ. Likewise, if warming is not uniform throughout an organ, thermal gradients develop that can cause cracks in the organ,^[^ ^9^, ^10^, ^11^ ^]^ much like the cracking of an ice cube when its exterior temperature is substantially different from its internal temperature (**Figure** **1**). It is noteworthy that as the size of the organ increases, the geometric limitations of heating make rapid and uniform warming even more challenging.

In order to overcome the challenges of rapid and uniform rewarming, our team developed a method for volumetric warming of vitrified systems termed “nanowarming.”^[^ ^8^ ^]^ Nanowarming uses radiofrequency (RF) excitation‐based heating of silica‐coated iron oxide nanoparticles (sIONPs), distributed within the biospecimen. The applied alternating fields induce rapid warming from magnetic hysteresis losses as the sIONPs are excited by the RF field, resulting in fast and uniform volumetric warming. One attractive aspect of nanowarming is that it can theoretically scale to human organ systems.

A brief review of some of the seminal contributions to this area follows‐ Etheridge et al., first presented proof‐of‐concept RF nanowarming in 1 mL CPA solution (in vitro) systems with theoretical modeling in scaled‐up cylindrical systems, illustrating the efficacy of nanowarming versus convective warming.^[^ ^12^ ^]^ Following this initial study, Manuchehrabadi et al. successfully demonstrated RF nanowarming experimentally in 1–80 mL CPA solution (in vitro) systems, and in biological systems, including human dermal fibroblast (HDF) cells (1 mL), aortic heart valve leaflets (1 mL), porcine carotid arteries rings and segments (in 1 and 50 mL volumes, respectively), and porcine femoral arteries (50 mL) with high post‐warming viability.^[^ ^8^ ^]^ Liu et al. showed that stem cells microencapsulated in hydrogels could be successfully vitrified and nanowarmed in low CPA concentrations and small volumes (0.25 mL) and recovered with high viability and retention of cellular function.^[^ ^13^ ^]^ More recently, Horie et al. showed that human induced pluripotent stem cells (and cell aggregates) could be successfully vitrified and rewarmed with high viabilities in scaled up 20 mL CPA systems using RF nanowarming.^[^ ^14^ ^]^ Using differential scanning calorimetry experiments, Xu et al. showed that the presence of magnetic nanoparticles in CPA suppresses ice nucleation and growth during cooling and rewarming.^[^ ^15^ ^]^ In parallel computational modeling efforts, Solanki et al. and Eisenberg et al. provide detailed analysis of the thermomechanical stresses during cooling and nanowarming in CPA solutions.^[^ ^16^, ^17^ ^]^ In efforts focused on nanoparticle development for nanowarming, Shore et al. presented the alternative use of ferromagnetic nanowires with higher SAR for rapid RF nanowarming of CPA solutions (≤ 0.5 mL).^[^ ^18^ ^]^ At the organ level, Gao et al. described a novel scaled up synthesis method for colloidally stable sIONPs exhibiting in vitro and ex vivo colloidal stability in CPA, and proof‐of‐principle loading and washout of sIONPs in kidneys.^[^ ^19^ ^]^ Chiu‐Lam et al. recently synthesized superparamagnetic iron oxide nanoparticles (SPIONs) which were colloidally stable in CPA, and then manually loaded them with CPA into rat hearts, followed by a physical demonstration that a cryogenically cooled heart could be nanowarmed.^[^ ^20^ ^]^ These reports show that iron oxide nanoparticles can be made colloidally stable in CPA and can be perfusion loaded and nanowarmed. However, careful characterization and validation of CPA and IONP perfusion loading and unloading, vitrification, uniform and rapid nanowarming to avoid crystallization and recovery of viability and function in an organ remains to be demonstrated.

In the current paper, we describe how we successfully nanowarmed a cryopreserved rat kidney with preserved morphology and good viability at the cellular level. This milestone is a key proof‐of‐concept along the way toward successful banking of cryopreserved human organs and their eventual use in regenerative medicine and ultimately transplantation. Specifically, we now demonstrate the following: 1) The ability to load CPA and sIONPs throughout the kidney by vascular perfusion; 2) The ability to cool faster than the CCR required to avoid ice; 3) The ability to rewarm faster than the CWR (to prevent ice) and with minimal thermal gradient (to prevent cracking); 4) The ability to unload both CPA and sIONPs from the kidney; and 5) Intact morphology and cellular viability of the cryopreserved and rewarmed kidney.

## 2 Results

### 2.1 Perfusion Loading of CPAs and sIONPs into Kidneys

**Figure 2:**
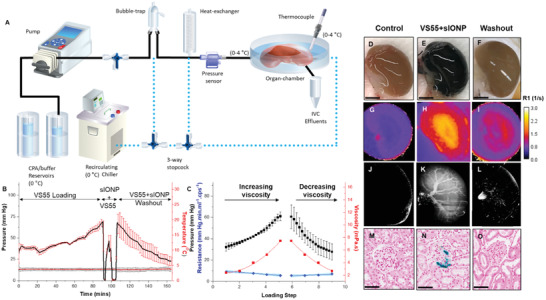
Hypothermic perfusion loading and unloading of rat kidneys with VS55+sIONPs. A) Schematic layout of the hypothermic perfusion circuit. The cold CPA (0–4 °C) is perfused using a peristaltic pump through a bubble trap and a heat exchanger before entry into the renal artery. Pressure and temperature sensors record the arterial pressure and chamber temperature, respectively, throughout the experiment. A circulating chiller is used to cool the bubble trap, heat exchanger, and organ chamber. B) Mean arterial pressure and chamber temperature variation over time during hypothermic perfusion loading and unloading of a rat kidney with VS55 and sIONPs. Error bars indicate the standard error (SEM) for *n* = 4 rat kidneys. The loading steps were Euro Collins (EC), 18.75%, 25%, 50%, 75%, and 100% v/v (8.4 m) VS55 and the wash out steps were 75%, 50%, 25% VS55 (v/v), and EC (Table S2, Supporting Information); no osmotic buffering was used during washout. C) Variation of peak pressure over the loading and unloading steps correlates with increase and decrease in viscosity of the perfusate, as VS55 concentration is increased during loading and decreased during washout. At each loading step, there is an overall increase in pressure due to the increase in viscosity overlapping with a transient reduction in pressure due to the vascular osmotic response to the increased concentration (osmotic vasodilation—Figure 2B; Figure S2, Supporting Information); As a net result, calculated resistance, *R* (blue points) actually decreases as viscosity increases; shaded blue region indicates 95% confidence interval of a linear fit of *R* versus time (loading steps). *R* is only calculated for VS55 loading and washout, not during sIONP loading. D–F) Gross images of a control, VS55+sIONP loaded and washed‐out kidney, respectively. Scale bar is 0.5 cm. G–I) MR images depicting the distribution of sIONPs, as based on the water relaxation rate constant (*R*1), of a control, VS55+sIONP loaded and washed‐out kidney, respectively. J–L) X‐ray µCT images of a control, VS55+sIONP loaded and washed‐out kidney, respectively, showing spatial resolution of sIONPs in the kidney vasculature. M–O) Prussian blue staining, to show Fe deposition in a control, VS55+sIONP loaded and washed‐out kidney, respectively. Fe localization is seen in the glomeruli of VS55+sIONP loaded kidneys. Washed out kidneys show clear glomeruli. Scale bar is 150 µm for histology images.

Perfusion loading of CPA and sIONP was performed using a custom‐built computer‐controlled perfusion device. System capabilities include multiple perfusion channels with flow dampening, bubble trap, mixing chamber, heat exchanger, and pressure and temperature monitoring and control as shown in **Figure** **2**A. VS55 was selected for the CPA in this study due to its well‐defined thermal properties including specific heat and density (*C*_p_ *, ρ*), known CCR, and CWR (Table S1, Supporting Information) and well‐characterized toxicity profile in kidney slices.^[^ ^7^, ^21^ ^]^ sIONPs were used as they have demonstrated biocompatibility and colloidal stability within VS55 for nanowarming applications.^[^ ^19^ ^]^ Perfusion was achieved through the renal artery where VS55 was loaded by incrementally increasing concentration while reducing temperature to 0–4 °C as noted in Table S2 in the Supporting Information. These 15‐minute steps reduce injury from osmotic and chemical stress while ensuring adequate time for CPA diffusion into the kidney tissue (Figure S2, Supporting Information). During perfusion the mean pressure increased but remained within the physiologic range (0–100 mm Hg) with a flow rate of 1.5 mL min^−1^ as shown in Figure 2B (for individual kidney arterial pressure line plots vs time, refer to Figure S1A in the Supporting Information). This rise is likely due to the viscosity increase of VS55 introduced in each perfusion step and can be appreciated by the linear correlation between viscosity and mean pressure as shown in Figure 2C (for correlation of pressure and viscosity, refer to Figure S1D in the Supporting Information).

Immediately following CPA loading, a colloidal mixture of 10 mg Fe mL^−1^ sIONPs in 100% VS55 (VS55+sIONP) was perfusion loaded into the kidneys at a constant rate of 0.5 mL min^−1^. During loading, the perfusion pressure increased, but remained in the physiologic range (40–80 mm Hg) as shown in Figure 2B (for loading profile, see Figures S1 and S3B in the Supporting Information). Visual inspection of the kidneys during VS55+sIONP colloid loading showed a gradual change from brown to solid black as the dark sIONP particles saturated the renal vasculature (Figure 2D,E). This clearly shows the distribution of sIONP within the renal cortex. However, to examine the distribution of sIONP throughout the vasculature of the kidney µCT imaging was used as shown in Figure 2K (Figure S3, Supporting Information). These images show HU contrast from sIONP within larger vascular structures (>29 µm resolution) within the kidney. Furthermore, the images also show an increase in overall diffuse background HU throughout the kidney which is consistent with loading within the capillaries. To further examine loading at the capillary level, Prussian blue staining of histological slices was used. These showed blue staining (iron) especially within the glomerular capillary tufts (Figure 2N). The combination of µCT imaging and Prussian blue staining suggest that the sIONPs are loaded throughout the kidney within vascular structures.

### 2.2 Vitrification of Kidneys

Once kidneys were loaded with VS55 ± sIONP they were vitrified by cooling to cryogenic temperatures (−150 °C) in a controlled rate freezer (CRF) and then stored in a deep cryogenic freezer at −150 °C. More specifically, loaded kidneys were placed within polyethylene cryo bags also loaded with 20 mL of VS55 ± sIONP and transferred to a CRF for cooling (Figure S6, Supporting Information). The CRF was rapidly cooled at −40 °C min^−1^, a rate faster than CCR of VS55, from 0–4 °C to −121 °C which is just above the glass transition temperature (−123 °C for VS55^[^ ^8^ ^]^). Annealing above *T*_g_ allows temperatures to equilibrate throughout the kidney, thereby reducing thermomechanical stress, a driving force for cracks, before transitioning into the brittle vitrified state. After annealing for 25 min, the temperature was further decreased to −150 °C at −10 °C min^−1^ to fully vitrify. The kidney was then carefully placed in a −150 °C freezer prior to rewarming and further testing.

**Figure 3:**
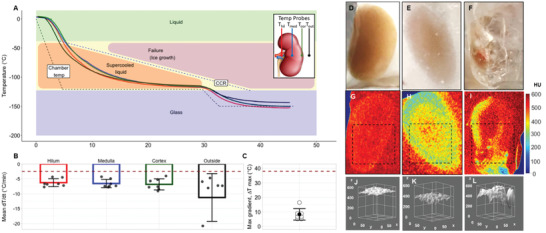
Vitrification success and failure in VS55 loaded kidneys. A) Temperature versus time (*T* vs *t*) plot during cooling of a kidney for vitrification. Cooling was performed in a bag‐setup (Methods, Figure S6, Supporting Information) in a controlled rate freezer (CRF). As shown in the inset, fiber optic temperature probes were placed in the hilum, medulla, cortex and outside the kidney (inside the bag) to measure temperature distribution during cooling. B) Mean, SEM, and scatter plot of cooling rates measured at each probe location, for *n* = 7 kidneys, relative to CCR of VS55 (dotted line). Mean cooling rates at all probe locations were faster than the CCR for VS55, suggesting no ice crystallization. C) Mean, SEM, and scatter plot of maximum gradient ∆*T* in the glassy state for *n* = 7 kidneys. These are well below the dotted line indicating the maximum stress‐to‐fracture threshold of *∆T*_max_ (38 °C) for VS55. D–F) Gross images of a vitrified, frozen, and a cracked kidney, respectively. G–I) X‐ray µCT of a vitrified, frozen and cracked kidney, respectively. X‐ray attenuation differences between cases, expressed in Hounsfield Units (HU), are used to detect amorphous (vitrified) versus frozen regions in the kidney and abrupt/sharp X‐ray attenuation changes, indicating cracks. J–L) 3D surface histogram plots for dotted square regions in Figure 3G–I, respectively, indicate spatial X‐ray attenuation differences in vitrified, frozen, and cracked kidneys, within a given plane.

**Figure 4:**
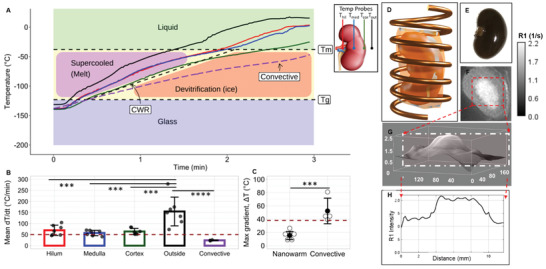
Rewarming of VS55 + sIONP loaded and vitrified rat kidneys. A) Temperature versus time (*T* vs *t*) plot during nanowarming and convective warming (negative control) of a kidney from the vitrified state. Nanowarming was performed by placing the vitrified kidney in an RF solenoid coil (D) and rewarming at 180 kHz and 63 kA m^−1^ alternating magnetic field. As shown in the inset in (A), fiber optic temperature probes were placed in the hilum, medulla, cortex and outside the kidney (fixed inside the vitrified bag) to measure temperature distribution during rewarming. Convective rewarming was performed in a water‐bath set to 37 °C (Figure S8, Supporting Information). B) Mean, SEM and scatter plot of nanowarming rates measured at each probe location, for *n* = 7 kidneys, relative to CWR of VS55 (dotted line). Mean rewarming rates at all probe locations were greater than the CWR, suggesting minimal likelihood of devitrification. ***p < 0.001, ****p < 0.0001 using one‐way ANOVA with Tukey's multiple comparisons. C) Mean, SEM and scatter plot of maximum gradient *∆T* in the glassy state for *n* = 7 kidneys during rewarming. ***p < 0.001, using a two‐tailed unpaired t test. Dotted line indicates maximum stress‐to‐fracture temperature difference threshold, *∆T*_max_ (38 °C). E) Gross image of a nanowarmed kidney. The dark contrast is from the sIONPs (Figure S4, Supporting Information). F) MRI map of *R*1 of a VS55+sIONP loaded kidney shows a higher concentration of sIONPs in the medulla relative to the cortex. G, H) 3D and 2D *R*1 contrast histogram plots, respectively to quantify relative sIONP concentrations.

Temperature monitoring within the kidney and surrounding media provides spatiotemporal temperature and cooling rate measurement (**Figure** **3**). The cooling shows an exponential response down to the annealing temperature (−121 °C) and subsequent cryogenic storage (−150 °C). The mean rates of cooling exceeded the CCR of VS55 (−2.5 °C min^−1^) between −45 and −90 °C, the maximum ice crystallization zone, as shown in (Figure 3B).^[^ ^7^, ^22^ ^]^ While the temperature profiles within the kidney tracked quite closely, the response within the surrounding bag dropped more quickly as shown in Figure 3A. For instance, the rates within the kidney were between −4 and −10 °C min^−1^, while the rates within the bag were −4 to −24 °C min^−1^ (Figure 3B). A further benefit of thermometry is the assessment of thermal gradients that can drive mechanical stress within the vitrified kidney. Fortunately, the mean gradient between the temperatures of −105 to −150 °C was 8.2 °C, well below the maximum tolerable thermal gradient (∆*T*_max_) predicted for VS55 (38 °C), defined by the “thermal shock equation” as shown by the dotted line in Figures 3C and **4**C and presented in the Supporting Information. Thus, the measured thermal gradients were within acceptable tolerance limits for both speed and uniformity during cooling, a finding confirmed by the observation that none of the kidneys recovered using nanowarming showed visible cracks (Figure 3D); unlike convective failures due to either slow cooling that resulted in ice (Figure 3E) or LN2 plunge cooling that resulted in catastrophic fracture (Figure 3F).

**Figure 5:**
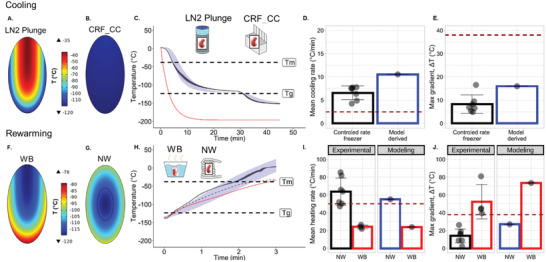
Computational thermal modeling of vitrification and rewarming of kidneys. A,B) Temperature distribution within a kidney approximated as an ellipsoid (2 cm x 1 cm x 1 cm), modeled at the coronal plane through the center for LN2 plunge (failure) and CRF convective cooling (success) cases, near *T*_g_ (Supporting Information). C) Numerical solutions showing temperature versus time (*T* vs *t*) plots for LN2 plunge (red = average of maximum and minimum cooling trajectories) and CRF convective cooling (blue shaded) relative to experimental plots (black‐mean and gray‐SD ribbon). The blue ribbons show spatial variation (range) in temperature within the kidney. Kidney cooling rate in CRF (black) was computed by taking the average of all temperature probes within a kidney, and then averaging this data over *n* = 7 kidneys plotted with SD gray ribbon. D,E) Bar plots summarizing modeled cooling rates and maximum gradients *∆T*, respectively, within the kidney, relative to experimental values (See Supporting Information for calculation). Black scatter‐plot of cooling rates, averaged over all probe locations for *n* = 7 kidneys, (from Figure 3B) are shown overlaying the experimental bar plots for reference. Maroon dotted line in (D) indicates CCR of 8.4 m VS55. Maroon dotted line in (E) indicates *∆T*_max_ threshold corresponding to stress‐to‐fracture limit, (38 °C) for VS55. CRF d*T*/d*t* (blue bar in (D)) was computed by taking the average d*T*/d*t* of the modeled maximum and minimum temperature rate limits across the modeled kidney volume. F,G) Temperature distribution within a kidney section taken through the center, for water‐bath convective warming (WB) and Nanowarming (NW) of a kidney, near *T*_g_. H) Numerical solutions showing temperature versus time (*T* vs *t*) plots for WB rewarming (red) and Nanowarming (blue shaded) relative to experimental plots (black solid lines and gray ribbons representing mean and SD for NW, respectively). The gray SD ribbons show spatial variation (range) in temperature within the kidney. The black dashed line represents experimental spatial‐mean WB rewarming temperature averaged over three temperature probes. I,J) Bar plots summarizing modeled rewarming rates and maximum thermal gradients *∆T*, relative to experimental values. Black scatter plot of nanowarming rates, averaged over all probe locations for *n* = 7 kidneys, (from Figure 4B) are shown overlaying the experimental bar plots for reference. In (I), experimental NW (black bar) was computed by taking the average of all temperature probes within a kidney, and then averaging this data over *n* = 7 kidneys. For modeling in (I), NW d*T*/d*t* was computed by taking the average of the maximum and minimum temperature rate limits across the modeled kidney volume.

To further confirm these experimental observations, finite element modeling of heat transfer within the kidney was performed using COMSOL based on a 3D ellipsoidal geometric approximation of the kidney (Figure S9, Supporting Information). Model‐derived cooling inside a CRF leads to more uniform temperature distribution and, therefore, smaller temperature gradients as compared to the convective cooling negative control (plunging the kidney into LN2) (**Figure** **5**A–E). Modeling of the temperature gradient near the glass transition temperature shows large gradients as high as 80 °C during plunging (Figure 5A), which exceeds the stress‐to‐fracture temperature difference for cracking of 38 °C (Supporting Information). In contrast, CRF cooling simulation predicts cooling rates −10 °C min^−1^, faster than the CCR and lower temperature gradients in the range of 7–15 °C (Figure 5D,E), similar to values observed experimentally. The low gradients in the glassy state are achieved by both the annealing step at −121 °C (just above *T*_g_) and the slow cooling rate (−10 °C min^−1^) thereafter as the kidney entered the glassy state. Modeling shows the expected maxima and minima temperature versus time within the kidney (Figure 5C, blue shaded region) and compares favorably with the experimental results (Figure 5C, gray shaded region).

Vitrification success as well as crystallization and cracking failures within kidneys were directly visualized by eye and further confirmed by µCT imaging as shown in Figure 3. The cooling protocol shown in Figure 3A (Supporting Information) yields a kidney with glassy appearance on gross visualization (Figure 3D), high radiodensity (i.e., expressed as Hounsfield Units, HU > 500) on µCT imaging (Figure 3G), and a smooth 3D surface histogram of HU across a µCT cross section (Figure 3J). These findings suggest that the kidney is vitrified without crystallization or fracture planes. In contrast, Figure 3E,H,K shows a slow‐cooled (frozen) kidney, where X‐ray attenuation drops into the 300–400 HU range, with significant spatial heterogeneity (up to 200 HU across a single cross‐sectional plane). Finally, Figure 3F,I,L shows a cracked kidney, where sharp transitions in X‐ray attenuation are observed due to cracks or ice regions, with observable differences as large as 400–500 HU within a single cross‐sectional plane. Previous work has demonstrated the ability to distinguish between crystalline versus amorphous phases in biological tissues at cryogenic temperatures based on X‐ray attenuation (Supporting Information). Through the use of VS55 controls, a calibration scale of HU versus VS55 phase was generated (see the color scale in Figure 3G–I where HU > 500 (red‐orange) corresponded to a vitrified state, whereas 200–500 HU (yellow‐green) corresponded to the presence of significant ice in the system). Abrupt spatial transitions in radiodensity corresponded to the presence of cracks detected by a 3D surface histogram representation of a sectional plane showed relative spatial uniformity of the phase within that cross‐sectional plane (Figure 3J–L). These findings confirm our ability to vitrify a VS55 loaded kidney as well as our ability to measure failure modes of crystallization and cracking within a VS55 loaded kidney.

### 2.3 Nanowarming of Kidneys without Devitrification or Cracking

Nanowarming allows rapid and uniform volumetric heating of VS55+sIONP loaded kidneys. Figure 4A,B shows 4‐point thermometry‐based temperature versus time measurements during nanowarming. The mean heating rates at all measured points within the kidney during nanowarming are between 55 and 65 °C min^−1^, which exceeds the CWR threshold for VS55 of 50 °C min^−1^. The average maximum gradient (∆*T*_max_) achieved within the kidney in a glassy state during nanowarming is 15.3 °C, which is well below the ∆*T*_max_ corresponding to the stress‐to‐fracture temperature limit of VS55‐glass (38 °C) (Figure 4C). Most importantly, all kidneys were recovered intact after nanowarming without any visible cracks. Figure 4E shows the gross image of such a nanowarmed kidney. In contrast, convectively (water‐bath) warmed kidneys formed large cracks causing the kidney to physically break apart during rewarming (Figure S8, Supporting Information). In addition, this convective warming led to lower rates of warming below the CWR of VS55 (Figure 4A, purple dashed line). In short, convective rewarming led to both large thermal gradients and warming rates lower than CWR (dashed line in Figure 4C, 38 °C), resulting in physical damage to the kidney by cracking and crystallization (Figures 4C and 3F).

In order to model nanowarming, the concentration of the iron within the tissue is needed to estimate the volumetric heating. Sweep imaging with Fourier Transformation (SWIFT) magnetic resonance (MR) image analysis of a VS55+sIONP loaded kidney (Figure 4F–H) was used to determine relative concentrations of iron in different regions in the kidney. Using the grayscale 3D histogram and line plot as shown in Figure 4G,H, the concentration within the medulla was estimated as two‐fold higher than cortical concentrations. The total Fe in the kidney (≈2 mg) was determined previously by ICP‐OES of kidneys loaded by the same protocol^[^ ^19^ ^]^ (Supporting Information—Computational Modeling). This allowed the mass of Fe (mg Fe g^−1^) in the medulla and the cortex to be determined and the heating rates to be directly estimated (from Specific Absorption Rate (SAR) in W mg^−1^ Fe) (Figure S9, Supporting Information) for use in the nanowarming model. Results shown in Figure 5F,G,J show the model‐derived temperature gradients near *T*_g_ during rewarming for nanowarming versus water bath convective control. Nanowarming shows rapid rewarming with lower than 25 °C thermal gradients across the kidney in the glassy temperature region. In comparison, convective water bath rewarming yields larger than 40 °C thermal gradients across the kidney. Additionally, the lower region of the convectively warmed kidney heats faster than the upper region due to that region's spatial proximity to the convective boundary (water bath‐bag interface) at the bag edge, where the kidney rests. The blue band in Figure 5H shows the variation of modeled temperature across the kidney falls within the experimentally measured 4‐probe temperature data (gray shaded region). The model‐derived gradients agree with experimental results: ∆*T*_max_ = 15.3 °C for nanowarming and ∆*T*_max_ > 40 °C for water‐bath. Overall, the model predicts that the nanowarmed kidneys rewarm at rates above CWR of VS55 while convective rewarming yields low heating rates (<CWR) in some regions and unacceptable thermal gradients (Figure 5I).

All the factors described in Figures 4 and 5 may be affected by the sample volume, but in principle, nanowarming can be successfully scaled even to large volumes. To demonstrate this, a preliminary proof of principle study was undertaken using a larger rabbit kidney model. The rat kidney mass is in the range of 1–4 g, whereas the rabbit kidney mass is normally in the range of 12–15 g, and in these studies, warming was undertaken with a total sample mass of 80 g (kidney plus surrounding solution). For these studies, we used a different CPA, M22, which was first loaded into three rabbit kidneys, essentially as previously described.^[^ ^23^ ^]^ A simpler and earlier type of iron oxide nanoparticle system (EMG‐308) was mixed with M22 at 20 mg Fe mL^−1^, filtered coarsely and then through a 0.22 µm filter (final concentration estimated as 14 mg Fe mL^−1^), and perfused into the rabbit kidney at various times (3, 15, and 20 min) after beginning perfusion with M22 alone. Although EMG‐308 tended to aggregate and slow M22 distribution if added too soon, all kidneys turned black rapidly and uniformly upon perfusion, the ureters showed darkening as well, and we found that introducing the EMG‐308 after 20 min of prior M22 perfusion enabled it to be successfully distributed along with M22 throughout the kidney.^[^ ^23^ ^]^ Vitrification then took place with the kidney immersed in an M22 solution whose EMG‐308 content was diluted to 3–4 mg Fe mL^−1^ to account for the effect of vascular confinement of the nanoparticles on the mean intra‐renal EMG‐308 concentration, and the kidneys were stored at −145 °C prior to rewarming. To test nanowarming, the vitrified kidneys were placed in a 15 kW RF coil and rewarmed at 180 kHz, 63 kA m^−1^ with thermal monitoring using two fluoroptic thermal probes placed within the kidney. The warming curves for the most successfully loaded kidney show rates of warming of 55 °C min^−1^, which exceeds both the CWR for VS55 and M22 (<1 °C min^−1^)^[^ ^23^ ^]^ with a minimal thermal gradient (Figure S10, Supporting Information). The two other kidneys both rewarmed at rates above the critical warming rate of M22 (data not shown). Convective warming of the same kidney, within its 80 mL total volume, showed gradients approaching 40 °C, again showing that nanowarming is able to rewarm far more uniformly than convective heating. The difference between nanowarming and convective heating is expected to be even larger when scaling to human sized organs. Unlike convection, volumetric heating during nanowarming is expected to be independent of heating volume, provided uniform nanoparticle perfusion and RF fields of the same magnitude and uniformity can be maintained. EMG‐308, a simpler and earlier type of nanoparticle, was only used for the rabbit kidney nanowarming studies. For all other studies, sIONPs were used as they are colloidally very stable compared to EMG‐308,^[^ ^19^ ^]^ and therefore, will ensure uniform perfusion. However, one important caveat during scaling to human‐sized organs is that we would have to account for non‐uniform power deposition from eddy‐current heating. These currents are predictable and the resulting power deposition from them increases with distance (squared) from the center of the coil. Previous estimates on a 10 cm diameter system (large enough for human organs) suggest a maximum effect at full radius of 20% power deposition from eddy current to sIONP heating.^[^ ^8^ ^]^ However, with proper field and rewarming protocol design, these effects can likely be controlled within an acceptable range of non‐uniformity even in large organ systems.

### 2.4 Washout of CPAs and sIONPs from Kidneys

Following rewarming or after control loading only, both CPA and sIONP need to be unloaded from the kidneys. Unloading was performed by further perfusion with stepwise reduction in CPA concentration back to baseline perfusate. sIONP were washed out by passive unloading along with CPA. As noted above, in Figure 2B, Figures S11A and S2A (Supporting Information), each step demonstrates a transient peak in pressure likely related to capillary equilibration, followed by a more gradual decline as the system equilibrates with the perfusate in each step, and an overall decline as the VS55 concentration (viscosity) reduces to that of the carrier buffer EC alone.^[^ ^24^ ^]^ Similar to the loading process, these transient peaks are attributed to the kidney vasculature and parenchyma adapting to osmotic changes in the perfusate (Figure S2, Supporting Information). A reversal of accumulated osmotic vasodilation can be detected by the slow rise in the intrinsic resistance of the kidney as CPA concentrations approach zero and only the buffer (EC) is left (Figure 2C).

In Figure 2E–N, the washout process in kidneys was characterized following loading of VS55+sIONPs (without nanowarming). First, the renal vein effluents were confirmed to be clear (with no visible sIONPs) (Figure S4B, Supporting Information), and the pressure slope approached zero (Figure 2C). Visual examination of the kidneys showed that the washed‐out kidney changed from dark black to brown, to light brown (Figure 2E,F) suggesting at least cortical clearance of most of the sIONPs. However, a slightly darker color than the control (Figure 2D) suggests some retention of sIONPs in the kidney (also seen in the gross hemi‐section of the kidney in Figure S3G,H in the Supporting Information. Further assessment of residual sIONP was carried out through MR T1 relaxation and derived *R*1 maps as shown in Figure 2G–I. These images show differential Fe loading in the cortical versus medullary/juxta‐medullary regions, with a roughly 2× higher average concentration in the medullary region, as indicated by *R*1 contrast (Figure 2H) which has been shown previously to scale linearly with Fe concentration.^[^ ^25^, ^26^ ^]^ Compared to the control kidney, the washed‐out kidney exhibited minor Fe (sIONP) retention in the medullary region and largely clear cortical regions as indicated by the comparable *R*1 contrast to the control (Figure 2G vs 2I). While the higher sIONP retention in the medullary region is ≈25% above control, it is much lower (about four‐fold) than the fully VS55+sIONP‐loaded kidney based on *R*1 signal. The total iron concentration, cFe, in these washed out kidneys was found to be ≈0.0024 mg mg^−1^ dry weight tissue (Figure S3, Supporting Information, *n* = 3). The loading and unloading of sIONP was further characterized by µCT (Figure 2J–L). In VS55+sIONP‐loaded kidneys (Figure 2K), contrast from radiopaque sIONPs was observed in major vessels. In washed‐out kidneys (Figure 2L) this contrast from sIONPs is largely absent except for in what appear to be cortical glomeruli (bright spots) and medullary rays and the vasa‐recta. A closer examination of the kidneys through histopathology and Prussian blue staining (stains Fe deposition) (Figure 2M–O) indicated that Fe was present in some glomeruli in the juxta‐medullary cortical regions and vasa recta capillaries in VS55+sIONP‐loaded kidneys (Figure 2N). However, Prussian blue staining was unable to identify sIONP in the cortical glomeruli in the washed‐out kidneys (Figure 2O; Figure S5, Supporting Information). Following nanowarming, kidneys exhibited similar washout characteristics and Fe clearance as evidenced by Prussian Blue staining (Figure S11, Supporting Information).

### 2.5 Nanowarmed Kidneys show Preserved Viability, Architecture, and Intact Endothelium

**Figure 6:**
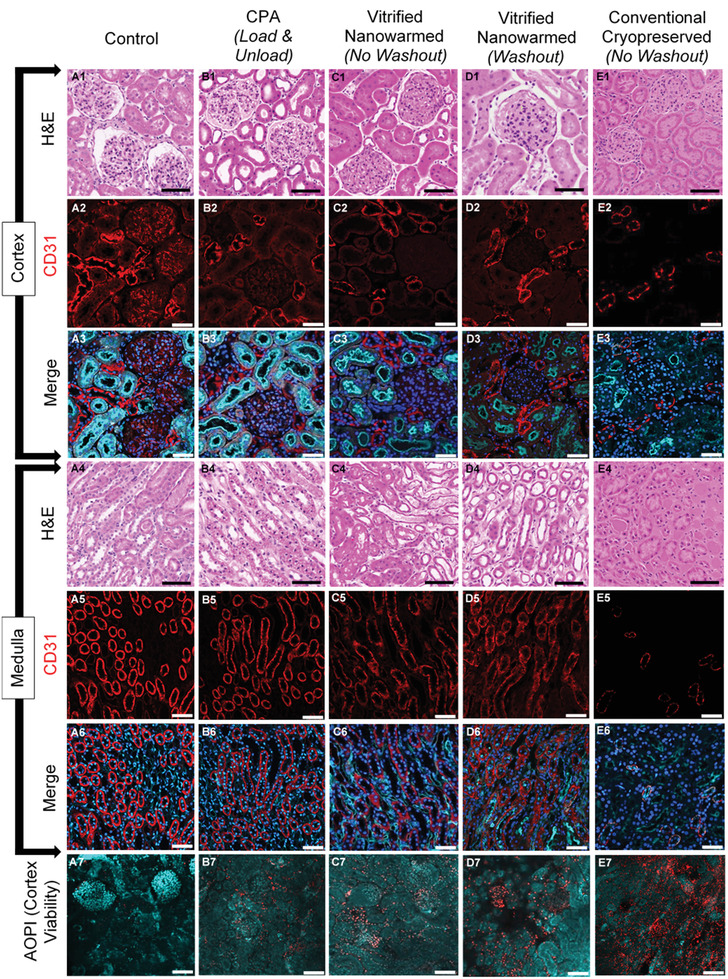
Histology, viability, and endothelial morphology of the kidney after experimental procedures. A1–E1, A4–E4) H&E photomicrographs to show renal cortex and medulla respectively, comparing morphological and architectural changes with CPA (VS55) perfusion, vitrification and washout following vitrification and nanowarming to fresh control and cryopreserved negative control sections (20×). A2–E2, A5–E5) Confocal microscopy shows vascular endothelial (CD31) labeling of kidney cortex and medulla and demonstrates changes to vascular endothelium across treatment groups (20×). A3–E3, A6–E6) Merged confocal microscopy of kidney cortex and medulla respectively, labeling vascular endothelium (CD31, red), nuclei (DAPI, blue), and d‐galactosyl residues of n‐acetyl‐d‐galactosamine end groups (Isolectin GS‐IB4, cyan) to demonstrate nuclear, vascular endothelium and tubular luminal alterations following preservation and rewarming (20×). A7–E7) Confocal microscopy demonstrates viability of kidney cortex by labeling live cells (Acridine Orange, cyan) and dead cells (Propidium Iodide, red) to assess viability at the center of a 500 µm thick kidney slice prepared from whole organs after each of the treatments (20×). Scale bar is 100 µm for confocal microscopy images (CD31 and AO/PI) (A2–E3, A5––E7) and 150 µm for histology images (A1–E1,A4–E4).

Post nanowarming and washout, the kidney and various controls were assessed for viability and structure. Histologic examination of renal cortex and medulla following CPA loading alone, nanowarming, and convective rewarming was performed. In general, the cortical and medullary architecture changes following CPA loading with VS55 were relatively minor compared to control (compare **Figure** **6**A1/A4–C1/C4), with some shrinkage of Bowman's space in the glomeruli and prominence of the tubular lumen in the cortex and medulla. Interestingly, there were largely similar histological and morphological characteristics following nanowarming compared with CPA load/unload alone (Figure 6D1/D4). In contrast, the convectively rewarmed kidney morphology showed significant architectural disruption with loss of interstitial space, shrinkage of Bowman's space, swelling of the tubular epithelial cells, and eosinophilic deposition in the tubules, lumen and interstitial spaces (Figure 6E1/E4). To assess the preservation of the vascular endothelium, confocal imaging and anti‐CD31 immunofluorescence antibody labeling was undertaken on nanowarmed kidneys and controls (Figure 6, rows 2 and 5). CPA loading and nanowarming slightly reduce the intensity of CD31 staining on vascular endothelium of both the cortex (C2, D2) and medulla (C5, D5), but the endothelial lining remains intact, and staining is well preserved compared to convectively rewarmed control kidneys (E2,5) where significant endothelial damage was apparent. In cases where higher arterial pressures occurred during perfusion and washout, greater cortical and medullary endothelial damage was observed (Figure S3R–U, Supporting Information). Finally, viability assessments with AO/PI demonstrated well‐maintained viability (teal cells) for both CPA only (Figure 6, B7) and nanowarming (Figure 6, D7) in comparison with fresh control (Figure 6, A7). However, some increased cell death (red cells) were noted in the glomeruli compared to elsewhere in the kidney for nanowarming and VS55 load/unload versus fresh control. In contrast, viability was significantly reduced in convectively warmed kidneys (Figure 6, E7), demonstrating widespread cellular damage across the nephron.

In summary, microscopic examination showed some morphologic changes associated with VS55 loading and unloading, but the addition of vitrification and nanowarming led to little further injury with respect to most of the endpoints examined. Each of these conditions was far better than convective rewarming. These data suggest that CPA toxicity from VS55 may be the main limiting factor for future functional assessments of this approach for kidney cryopreservation.

## 3 Discussion and Conclusion

Vitrification is currently believed to be the only process by which organs can potentially be stored up to months and potentially years at cryogenic temperatures without damage from ice crystallization.^[^ ^7^ ^]^ In this vitrified state, the metabolic activity of the cells effectively ceases, thereby reducing damage from ischemia. The glassy state results from an exponential increase in viscosity of concentrated solutions of CPA during cooling, largely precluding ice nucleation and growth during cooling and essentially arresting molecular diffusion during storage.

While vitrification has been demonstrated to be reproducibly successful, especially in small volume systems (≤3 mL) such as stem cells and embryos, and to be reasonably successful in thin vascular grafts,^[^ ^10^ ^]^ tissue sections,^[^ ^27^ ^]^ and even 12–15 g rabbit kidneys rendered unusually resistant to ice formation with M22,^[^ ^23^ ^]^ warming of rabbit kidneys and larger systems has remained an enduring problem. This relates to the dual needs for speed and uniformity of rewarming to avoid damage. For instance for VS55, rates > CWR of 50 °C min^−1^ and thermal gradients ≤ 38 °C are needed to avoid ice crystallization and cracking regardless of the size of the system,^[^ ^12^, ^22^ ^]^ and both become physically impossible to achieve simultaneously by external conduction warming as system volume increases beyond about 3 mL.

Direct electromagnetic rewarming methods such as microwave or dielectric rewarming have been previously attempted on cryopreserved organs. Rajotte et al and Ecker et al demonstrated the use of 2450 MHz microwave exposure to rewarm frozen kidneys.^[^ ^28^, ^29^ ^]^ These methods demonstrated novelty and some success but suffered from non‐uniform heating due to the small penetration depth at this wavelength and formation of standing waves in the cavity. Pegg and coworkers showed that dielectric heating of tissues in the frequency range of 300–1000 MHz was optimal for better penetration depth and uniformity of heating and further investigated the effects of CPA concentration and sample shape on the uniformity of heating.^[^ ^30^, ^31^, ^32^ ^]^ Evans and coworkers showed that dielectric rewarming results in a compromise, where either non‐uniformity in heating is accepted at higher frequencies or low rewarming rates are inevitable at lower frequencies. In addition to these issues, they describe factors that guide choice of frequency and applicator geometry in a resonant EM applicator to control “hot spots” (“thermal runaway”) and generate uniform heating.^[^ ^33^, ^34^ ^]^ Even with the use of uniform fields, inhomogeneous heating is expected due to variations in the dielectric properties of water versus non‐water tissue components (proteins, lipids, etc.), temperature dependence of the electrical permittivity and geometry of the sample.^[^ ^30^, ^31^, ^32^, ^33^, ^34^ ^]^ These non‐uniformities are further accentuated as the size is scaled up to the liter volumes that would be needed for human organs. Ruggerra et al previously described the use of RF helical coils in pressurized vessels for rewarming vitrified CPA under high‐pressure conditions at an average warming rate of 20 °C min^−1^/100 W per 100 mL.^[^ ^35^ ^]^ To avoid devitrification at these rewarming rates, however, high CPA concentrations (>9 m) are required, which can result in significant toxicity. Gao and coworkers describe an EM rewarming method using a resonant cavity and use of both electric and magnetic rewarming to get high rates (>200 °C min^−1^) in tens of milliliters of cryopreserved samples, however, scale up to organs was not demonstrated.^[^ ^36^ ^]^

Previously, our team showed successful rewarming and recovery of HDF cells and, porcine carotid and femoral arteries, in systems up to 50 mL (with physical scale up to 80 mL and feasibility analysis up to 1 L) using our novel nanowarming technology.^[^ ^8^ ^]^ In contrast to tissue nanowarming, where CPA distribution and rewarming are diffusion‐limited,^[^ ^37^ ^]^ one can leverage the vasculature of the organs to distribute the CPA+sIONPs and vitrify an organ as previously reported.^[^ ^7^, ^23^ ^]^ More recently, we and others have demonstrated advances in iron oxide nanoparticles and CPA+nanoparticle formulations that demonstrated in vitro heating rates > CWR, and in vitro and ex vivo colloidal stability that would allow translation to organ nanowarming.^[^ ^19^, ^20^ ^]^ In their recent work, Chiu‐Lam et al demonstrated thorough characterization of in vitro stability, heating rates and biocompatibility of their colloidally‐stable PEGsilane/APS coated superparamagnetic iron oxide nanoparticles (SPIONs), for physical nanowarming in rat hearts, highlighting the importance of the nanoparticle properties for this application. Additionally, they innovatively demonstrated the use of magnetic particle imaging (MPI) for quantification of the average iron concentration in SPION‐loaded and washed‐out hearts.^[^ ^20^ ^]^ The results presented by them are an important step in demonstrating physical nanowarming in hearts. However, to characterize the success or failure of perfusion, vitrification and nanowarming in an organ, a detailed ex vivo characterization during and following perfusion (perfusion pressure, high resolution imaging with contrast and spatial resolution), vitrification and nanowarming (thermometry and phase change) and biological assessment is warranted.

The present work addresses the limitations of convective rewarming by distributing sIONP heat sources along with CPA within and around the organ to allow volumetric uniform rewarming. This study was designed to test the plausibility of this concept by characterizing ex vivo: 1) ability to load and unload CPA+sIONPs with sufficient distribution of the sIONPs throughout the organ, 2) achievement of sufficient rates to vitrify and rewarm the organ without ice crystallization and/or cracking, and (3) the ability to recover the nanowarmed organ with comparable biological readouts compared to CPA load and unload controls. The outcomes of these inquiries have been mostly positive, and now, for the first time, we demonstrate application of nanowarming to reproducibly vitrify, rewarm and recover rat kidneys without ice crystallization or cracking, with preserved viability, architecture, and integrity of the endothelium as compared to loading and unloading controls.

Loading and unloading of CPA and sIONPs was achieved by perfusion of kidneys with VS55+sIONPs under controlled temperature and pressure (Figure 2A,B; Figure S1, Supporting Information). Step‐times of 15 min were chosen to allow sufficient time for diffusion of CPA from the blood vessels to the parenchyma (Figure S2, Supporting Information) while minimizing CPA exposure toxicity (Figure 6). sIONP distribution (uniformity) and washout (retention) were assessed by using a combination of imaging modalities (gross, MR, and µCT), histology and analytical methods (ICP‐OES) (Figure 2D–O; Figures S3, S5, S11, Supporting Information). These data, combined with diffusion time calculations (Supporting Information), Krogh modeling (Figure S2, Supporting Information), and calculated resistance (Figure 2C) suggest that the kidneys are fully loaded with VS55+sIONPs (10 mg Fe mL^−1^), (≈0.0163 mg Fe mg^−1^ dry weight^[^ ^19^ ^]^), and that they demonstrated efficient washout, with cFe ≈ 0.0024 mg Fe mg^−1^ dry weight. This concentration is comparable to remnant concentrations of Fe observed in kidneys in mice 24 h post i.v. (0.18 mg g^−1^) injection of iron oxide nanoparticles in a previous study,^[^ ^26^ ^]^ which were shown to be tolerable by the mice for at least a month. Additionally, these nanoparticles, at similar human equivalent doses (e.g., 14.6 mg kg^−1^) have been used in clinical applications such as MRI imaging.^[^ ^38^ ^]^ Moreover, as demonstrated in Figure 2, the use of sIONPs as contrast agents with existing clinical imaging modalities such as µCT and echo‐based MRI, and more recently, echo‐free methods like SWIFT MRI, allow imaging of a broad range of iron concentrations (from 0.1 to 10 mg Fe mL^−1^ and higher), thus allowing for image‐guided planning for nanowarming in future clinical applications.

Once loaded, the ability of the organ to cool to the vitrified state and rewarm at sufficiently rapid and uniform rates for the CPA of choice is critical and was assessed by both experimental and modeling approaches. During cooling, CPA loaded systems encounter the following temperature zones/physical states: (i) liquid, where *T* > *T*_m_; ii) supercooled‐liquid, between *T*_m_ and *T*_g_ (where the likelihood of ice crystallization is maximum in the −45 to −90 °C range);^[^ ^7^, ^22^ ^]^ and iii) amorphous glass, where *T* < *T*_g_ (Figure 3A; Supporting Information). Therefore, our efforts focused on creating a reproducible cooling protocol by achieving cooling rates > CCR in the region between *T*_m_ and *T*_g_, and by reducing thermal gradients in the region where *T* < *T*_g_ (as noted in CRF protocol and Figure S6 in the Supporting Information). During rewarming, the need for rapid and uniform temperature change is even more important. The CWR is generally >> CCR in order to outrun ice often seeded upon cooling that grows quickly upon rewarming.^[^ ^27^ ^]^ These rates, >50 °C min^−1^ for VS55, were achieved after VS55+sIONP loading and RF field exposure as shown by experimental measurement (Figure 4) and modeling (Figure 5; Figure S9, Supporting Information). Although 10 mg Fe mL^−1^ was perfused throughout the kidney, it was confined to the vasculature of the organ, leading to an effective/average iron concentration throughout the kidney (including vascular and non‐vascular regions) of ≈2.5 mg Fe mL^−1^. Thus, to minimize thermal gradients for *T* < *T*_g_ during rewarming, the sIONP concentration (cFe) in the surrounding VS55+sIONP solution was lowered to 4 mg Fe mL^−1^ to better match the heating within the kidney and to stay well below the stress‐to‐fracture threshold for VS55 (between the kidney and surrounding fluid). Close examination of the temperature versus time profiles demonstrates that some instantaneous warming rates do transiently drop below the CWR (≈40–50 °C min^−1^), especially in the cortex (Figure 4A; Figure S7, Supporting Information). Fortunately, these lapses generally occur at temperatures above −60 °C, where ice growth rates are relatively slow,^[^ ^39^ ^]^ and by application of ice crystallization kinetics modelling^[^ ^22^ ^]^ the worst case ice fraction is predicted to be ≤ 5–7% (Supporting Information). Future work can further reduce the potential for small amounts of ice by increasing warming rates or by using different vitrification solutions with greater glass forming potential.^[^ ^21^ ^]^ Warming rates can be increased by using either higher concentration sIONP or higher SAR sIONPs, as recently reported.^[^ ^20^, ^40^ ^]^ Warming rates can also be increased by adjusting the frequency or field magnitude of the applied RF field. For instance, here we used 15 kW (63 kA m^−1^, 180 kHz), but future work is planned on a new higher volume and higher power system at 120 kW (33 kA m^−1^, 360 kHz) which may increase the SAR by almost 50%. (SAR increases linearly with frequency in the 100 kHz–1 MHz range but starts saturating at H ≈ 35 kA m^−1^). Finally, a better glass forming CPA, like M22, with CCR and CWR < 1 °C min^−1^,^[^ ^23^ ^]^ combined with sIONPs at similar concentrations (10 mg Fe mL^−1^, volume fraction < 0.1%) will promote ice‐free vitrification and nanowarming, even when scaled to human‐sized organs (when combined with the aforementioned 120 kW (2.5 L VOI) AMF Life Systems nanowarming system). A proof of principle demonstration of the ability to physically nanowarm a M22+iron oxide nanoparticles loaded in a rabbit kidney is shown in (Figure S8, Supporting Information). Although M22 has a low CWR, its inability to fully permeate into perfused kidneys results in a need for much higher warming rates similar to those reported here.^[^ ^23^ ^]^

While the principal focus of this study was on physical demonstration of vitrification and rewarming of whole rat kidneys, we did perform an initial examination of biological endpoints. Specifically, we found that CPA loading and unloading (VS55 with or without sIONP) resulted in limited histologic changes (reduction in Bowman's space, decreased immunofluorescence staining intensity for the endothelial marker CD31, and the appearance of relatively small numbers of dead cells) in comparison to fresh control organs (Figure 6; Figure S11, Supporting Information). After VS55+sIONP washout, histological changes were intensified, but perfusion pressures remained within the physiologic range with vascular resistance similar to fresh kidneys (Figure 2B; Figure S1, Supporting Information). The maintenance of perfusion flow rates, low pressure, and low resistance suggests predominant preservation of vascular integrity, and such measures are important indicators of organ quality in current clinical practice.^[^ ^41^ ^]^ In contrast, convection rewarming led to significant alterations in morphology, endothelial integrity, and increased cell death (Figure 6; Figure S11, Supporting Information). Perhaps most importantly, vitrified and rewarmed kidneys were essentially the same as VS55 load and unload only, suggesting the main mediator of the observed damage was CPA toxicity and not vitrification and nanowarming.

Fortunately, CPA toxicity can be controlled in future studies by improved perfusion methodology^[^ ^21^, ^23^, ^42^ ^]^ and the use of less toxic and more stable vitrification solutions.^[^ ^21^ ^]^ Therefore, combining the enabling studies reported here with continually evolving perfusion and cryoprotection technology holds the promise of fully successful organ cryopreservation in the foreseeable future, with potentially revolutionary implications for the future of organ transplantation.

## 4 Experimental Section

### Study Design

The objective of this study was to evaluate the efficacy of Nanowarming technology in rewarming vitrified rat kidneys without damage from ice crystallization and cracking. The criteria for prevention of ice (CWR) and cracks (maximum temperature gradients) in the system were clearly defined prior to the study. Thermometry from nanowarmed kidneys was compared against appropriate convective rewarming controls—water bath rewarming. The arterial pressure criteria (*p* < 100 mmHg) during perfusion was also set prior to the experiments based on physiological pressure ranges in the rat kidney. Experimental repeats (*n* kidneys) are included in the captions of the figures. Rats were randomized for thermometry, biological and perfusion endpoints. Data from each individual kidney is included in Supporting Information. Biological endpoints comprising of cell viability, histological architecture and endothelium expression were compared against fresh harvested kidneys (positive controls) and convectively rewarmed kidneys (negative controls).

### Surgery and Cannulation

All animal experiments were approved by the Institutional Animal Care and Use Committee (IACUC) at the University of Minnesota (IACUC Protocol: 1905‐37029A). Male Sprague‐Dawley rats (Charles River Laboratories, Wilmington, MA) weighing 176–300 g were used in this study. General anesthesia was induced by 4% isoflurane and maintained with 1.5% of isoflurane and 1 liter‐per‐minute oxygen. Details on surgery are described in Supporting Information. Briefly, the infrarenal abdominal aorta was cannulated using a 20G bulb tip catheter (FTP‐20‐30, Instech Laboratories, Plymouth Meeting, PA) and secured using a 4‐0 silk ligature. A second catheter (male luer, 45518‐46, Cole Parmer, Vernon Hills, IL) was placed in the inferior vena cava for venous drainage. The suprarenal aorta and vena cava were ligated using silk ligatures and 10 mL University of Wisconsin (UW) solution containing 500 IU of heparin was perfused at 0–4 °C. The kidney was mobilized from the Gerota's fascia and kept in UW at 4 °C for further study.

### VS55+sIONP Perfusion Loading and Washout in Kidneys

CPA (VS55) and carrier solutions (EC) are freshly prepared before the experiments and stored in the refrigerator (0–4 °C) until use. A 10 mL syringe is filled with nanoparticle solution—10 mg Fe mL^−1^ sIONP in 100% VS55 and stored in the refrigerator. The composition of the CPA perfusate is summarized in Table S3 in the Supporting Information. Next, the perfusion circuit setup and the kidney connection protocol (Supporting Information^[^ ^23^ ^]^) are followed to connect the kidney for perfusion. Once the kidney is connected, pressure is stable and effluent is draining continuously, flowrate is increased to 1.5 mL min^−1^. Perfusion with EC is continued for 15 min to flush out the UW and establish a stable pressure baseline before introduction of CPA (VS55). Next, the pump inlet line is switched to the VS55 reservoirs, maintained at 0–4 °C (on ice), and VS55 is introduced in 15 min steps, where the concentration is progressively increased (v/v) in each step (Table S2, Supporting Information). During the final loading step (100% VS55), the syringe containing the VS55+sIONP (10 mg Fe mL^−1^) is primed, placed within the syringe pump and connected to a parallel tubing leading to the circuit outlet through a three‐way stopcock. This tubing was previously primed, leak/bubble tested and connected to the circuit outlet tubing via a three‐way stopcock. The flow rate of the syringe‐pump is set to 0.5 mL min^−1^ (constant, without turning on the flow). At *t* = 10 min during the 100% VS55 loading step, the syringe pump is turned on and simultaneously, the three‐way stopcock is switched to allow flow from the syringe pump to the circuit outlet tubing connected to the kidney, while blocking the flow from the main perfusion line (the peristaltic pump is turned off). The target loading volume is at least 2 mL (2× kidney vascular volume), thus targeting a time of 4–6 min, unless pressure >100 mm Hg, in which case the sIONP loading is stopped. Following VS55+sIONP loading, the kidney is disconnected for vitrification. For subsequent washout of VS55+sIONP, serial dilutions of VS55 in 1× EC are used in 15‐minute perfusion steps as indicated in Table S2 in the Supporting Information. Associated with each ex vivo perfusion experiment, a regular deviation (≤±2 mm) in the pressure is observed, because of the peristaltic action of the pump and noise in the pressure sensor measurement. However, estimation of the average arterial pressure in each experiment allows us to estimate the mean of the underlying VS55 loading‐unloading process, which is depicted with the standard error in Figure 2B.

### Vitrification of Kidneys

Before disconnecting the kidney from the perfusion circuit, a cryogenic Ziplock bag (McMaster‐Carr, Elmhurst, IL) is prefilled with 20–25 mL of cold VS55+sIONP solution (100% VS55 + 4 mg Fe mL^−1^ sIONP in1× EC) and maintained in a refrigerator at 0–4 °C. A Kryo560 (Planer, Middlesex, UK) controlled‐rate freezer (CRF) is used to controllably cool organs to cryogenic temperatures. Details on CRF protocol can be found in Supporting Information. Following the final perfusion loading step, the VS55+sIONP loaded kidney is disconnected from the circuit and cannulas are capped to prevent air‐bubbles inside. For the thermometry cohort, cryogenic fiber optic temperature probes (Qualitrol, Fairport, NY) are surgically placed inside the hilum, medulla, cortex and in the bag solution (outside the organ) and affixed using super‐glue (Gorilla Glue, Cincinnati, Ohio). The kidney vitrification protocol, discussed in detail in Supporting Information, is followed next. The vitrified kidney is stored in a −150 °C cryogenic storage freezer (MDF‐C2156VANC‐PA, Panasonic, IL).

### Nanowarming of Kidneys

Nanowarming was conducted in a custom‐built 15 kW AMF coil at 94% power (preset to 63 kA m^−1^ and 180 kHz). The coil and power system (AMF Life Systems, Auburn Hills, Michigan) are described in detail elsewhere.^[^ ^43^ ^]^ Prior to transport of vitrified kidney from freezer to coil, the temperature data‐logger (Qualitrol T/Guard, Fairport, NY) is started, and temperature is recorded at 1 s intervals. Temperature probes were placed prior to vitrification. The vitrified kidney insulated only by the cryobag is quickly (1 s) transferred from the cryogenic storage freezer (−150 °C) to the center of the solenoid coil (field variation ≤±5%) and the AMF is switched ON to initiate nanowarming. The AMF is switched OFF when the following criteria are satisfied in the given order: (i) The lagging temperature point, *T*_lag_ ≥ *T*_m_+10 °C, where *T*_m_ = melting point of VS55 (−38 °C) (ii) The leading temperature point, *T*_lead_ ≤ 0 °C. Generally, (i) takes priority over (ii), i.e., if (i) is satisfied, then target temperature for *T*_lead_ is −5 to 0 °C. The cryobag containing the kidney is recovered from the coil and placed over ice, where the kidney is removed from the bag and the temperature probes are disconnected. The kidney is transferred back to the perfusion chamber to proceed with VS55+sIONP washout.

### MR Imaging for sIONP Distribution

MRI imaging was performed in a single session. All MR images were acquired with a volume transmit/receive coil having an inner diameter of 3 cm (Varian, Palo Alto, CA) in a 9.4 T 31 cm bore magnet (Magnex Scientific, Yarnton, UK) interfaced to a research console (Varian, Palo Alto, CA). as described previously.^[^ ^19^, ^25^ ^]^ More details are provided in Supporting Information.

### µCT Imaging and Analysis for sIONP Distribution and Vitrified Kidneys

An optimized µCT set up was used to detect the vitrification of the kidney (Supporting Information). The samples were scanned in a µCT imaging system (NIKON XT H 225, Nikon Metrology, MI). The accelerating voltage was set 65 kV, and the current was set to 95 µA. The resolution was 0.029 mm for the room temperature scan and 0.061 mm for the vitrified state scan, respectively. The X‐ray attenuation is represented in Hounsfield units (HU), a clinical measurement which normalizes X‐ray attenuation values by the difference between those of water and air at 20 °C. The detailed setup for imaging^[^ ^44^ ^]^ is explained in Supporting Information.

### Histology and Confocal Imaging

Following hypothermic perfusion, the kidneys were cut in the coronal plane to expose the cortex and medulla for gross imaging, transferred to 10% Neutral Buffered Formalin and paraffin embedded within 48 h. Using a microtome, 5 µm sections were acquired for Hematoxylin & Eosin (H&E), Prussian blue and confocal microscopy. 40× magnification brightfield images were obtained (TissueScope LE, Huron Digital Pathology, St. Jacobs, Ontario). Sections were labelled with rabbit anti‐CD31 primary antibody (EPR17259, abcam, Cambridge, UK) and goat anti‐rabbit IgG H&L (AF‐647) secondary antibody (ab150083, abcam). The sections were also labelled with Isolectin Griffonia simplicifolia AF‐488 Conjugate (ThermoFisher, Waltham, MA) towards d‐galactosyl residues of Galactose *α*‐1,3 Galactose (Gal *α*‐1,3 Gal). Nuclei were labelled with 4’, 6‐diamidina‐2‐phenylindole (DAPI).

### 3D Computational Modeling

A computational finite element model consisting of a rat kidney, approximated as an ellipsoid, immersed inside a three‐dimensional bag, (Figure S9, Supporting Information) was used. The numerical heat transfer model, based upon the general non‐homogenous transient heat transfer equation, with volumetric heat generation term was used

(1) $$\rho C_{P}\;\frac{\partial T}{\partial t}=\;\nabla .\;\left[k\nabla T\right]+q_{v}^{{}^{\prime \prime \prime }}$$

where *ρ* is the density, *C*_p_ is the specific heat, *k* is the thermal conductivity, *t* is the time and *T* is the temperature in the region of interest in the system domain. The heat generation term$\;q_{v}^{{}^{\prime \prime \prime }}$ is computed based upon the concentration and the specific absorption rate (SAR_Fe_) of the sIONP (Equation S2, Supporting Information).^[^ ^45^, ^46^, ^47^, ^48^, ^49^ ^]^ The numerical solution to the above heat equation is solved in the commercial FEA code COMSOL Multiphysics 5.5 (Burlington, MA). For more details refer to Supporting Information.

### Statistical Analysis

At least four biological replicates were performed for all kidney perfusion and thermometry experiments. Kidney arterial perfusion pressure and temperature data is presented as mean ± SEM (*n* = 4 kidneys). Pressure data was collected every 0.5 s and was averaged over 1.5 s intervals (3 data points), to average the noise from oscillations in pressure arising from peristaltic action and noise in the pressure sensor measurement (≤±2 mm Hg). Arterial pressure requirement during perfusion was set as *p* < 100 mm Hg as part of the study design prior to the experiments, to stay within physiological kidney pressures and avoid damage to the vasculature. Thus, experiments where perfusion pressures exceeded 100 mm Hg were outliers and excluded from the study. VS55 viscosity at different concentrations was measured at 0 °C and presented as mean ± SD (*n* = 3 samples per concentration). Arterial pressure versus perfusate viscosity analysis was conducted by first identifying pressure peaks for each loading step relative to a linear piecewise baseline fit to the pressure data and by computing the first derivative of the pressure data. A weighted‐least squares polynomial fit was performed on the pressure peaks (Adj. *R*‐squared = 0.983) and intermediate values between peaks were determined through interpolation. The pressure peaks for each loading step were compared with viscosity in each loading step for correlation. Pearson's correlation coefficient (0.995), Adj. *R*‐square (0.987) and 95% CI were determined. Cooling and nanowarming were performed on *n* = 7 kidneys and presented as mean ± SEM. One‐way ANOVA with Tukey's Multiple comparisons test was conducted on thermometry data (rewarming rates) between regions in the kidneys and versus convective rewarming (Asterisks indicate statistical significance, ***p < 0.001, ****p < 0.0001). One‐way ANOVA with Tukey's Multiple comparisons test was performed on ICP‐OES washout data (*p < 0.05, **p < 0.005, ***p < 0.0005). For viability analysis/biological assessment using AO/PI in nanowarmed kidneys versus controls, one‐way ANOVA with Tukey's multiple comparisons test was performed (*n* = 4 kidneys per treatment group, *p < 0.05, **p < 0.005, ***p < 0.0005). A summary table of statistics is included in Table S7 in the Supporting Information. GraphPad Prism (GraphPad Software Inc, San Diego, CA), Origin (OriginLab Corp, Northampton, MA) and R ver 4.03 (Foundation for Statistical Computing, Vienna, Austria) were used to prepare plots and conduct statistical analysis.

## Conflict of Interest

The following patents are published—“Cryopreservative compositions and methods” U.S. Patent Application 14/775998 (Bischof, J.C., Etheridge, M.L., and Choi, J., University of Minnesota, 2016), “Mesoporous silica‐coated nanoparticles” U.S. Patent 10493098 (Haynes, C.L., Hurley, K.R., and Egger, S.M., University of Minnesota, 2019), “Cobalt‐iron nanowires for remote heating using an alternating magnetic field.” U.S. Patent Application 16/852850 (Shore, D.E., Gao, Z., Tabakovic, I., Bischof, J., and Stadler, B.J.H., University of Minnesota, 2020), “System and Method for cryopreservation of tissues.” International Publication Number WO 2020/150 529 A1 (Lee, C.Y., Bischof, J.C., Finger, E.B., Sharma, A., University of North Carolina at Charlotte, University of Minnesota, 2020, this is a provisional patent). All other authors declare that they have no competing interests.

## Author Contributions

Z.H., L.G., E.B.F., and J.C.B. contributed equally to this work. A.S. conceived and performed experiments and data analysis with support from J.C.B. and E.B.F. A.S. wrote the LabVIEW code for pump operation and real‐time pressure, temperature data logging, assembled, and characterized the perfusion circuit and performed all perfusion experiments and analysis. J.S.R. and B.N. performed rat surgeries and provided technical assistance in experiments. J.S.R. performed CD31 staining, confocal imaging, histology, and viability analysis. J.S.R. and A.S. performed data analysis on microscopy images. L.G. and A.S. performed computational modeling with support from M.E. Z.H. performed Krogh modeling, µCT imaging and reconstruction. Z.H. and A.S. performed analysis on µCT images. H.L.R. performed MR T1 imaging. H.L.R. and A.S. performed R1 image analysis. Z.G. and E.M. synthesized and characterized sIONPs. B.W. and G.M.F. performed rabbit kidney perfusion loading experiments. J.C.B. and E.B.F. acquired funding and J.C.B., E.B.F., and M.E. oversaw project administration. A.S. wrote the manuscript with support and input from J.C.B., E.B.F., J.S.R., L.G., G.M.F., and all coauthors.

## Supporting information

Supporting Information

## Data Availability

Data available in article supplementary material.
