# Supplementary material for: Vitrification and Nanowarming of Kidneys
Source: Adv Sci (Weinh). 2021 Aug 11;8(19):2101691. doi: 10.1002/advs.202101691 (PMC8498880; doi:10.1002/advs.202101691)
Supplement: Supplementary file 1 — Supporting Information [file ADVS-8-2101691-s001.pdf]

© 2021 Wiley-VCH GmbH

ADVANCED SCIENCE Open Access

Supporting Information

for *Adv. Sci.,* DOI: 10.1002/advs.202101691

Vitrification and Nanowarming of Kidneys

Anirudh Sharma, Joseph Sushil Rao, Zonghu Han, Lakshya Gangwar, Baterdene Namsrai, Zhe Gao, Hattie L. Ring, Elliott Magnuson, Michael Etheridge, Brian Wowk, Gregory M. Fahy, Michael Garwood, Erik B. Finger, John C. Bischof**

Supporting Information

Vitrification and Nanowarming of Kidneys

Anirudh Sharma[1], Joseph Sushil Rao[2], Zonghu Han[1†], Lakshya Gangwar[1†], Baterdene Namsrai[2], Zhe Gao[1], Hattie L. Ring[3], Elliott Magnuson[1], Michael Etheridge[1], Brian Wowk[4], Gregory M. Fahy[4], Michael Garwood[3], Erik B. Finger[2††], John C. Bischof[1,5††]**

Supplementary Materials and Methods

Surgery

General anesthesia through inhalation was induced in male Sprague-Dawley rats (Charles River Laboratories International, Inc), 7-10 weeks old, weighing 176 to 300 grams, with 4% of isoflurane, 1 liter-per-minute oxygen and maintained with 1.5% of isoflurane and 1 liter-per-minute oxygen. The anesthesia depth was confirmed with toe pinch reflex. The abdomen was prepared using 70% ethanol after clipping hair. A transverse abdominal incision was performed 5 mm below the xiphoid process and extended through a vertical midline incision to expose the abdominal cavity in a T fashion. The intestines and spleen were mobilized and retracted away from the left kidney and greater vessels. The abdominal aorta and inferior vena cava from the origin of left renal artery/vein down to the bifurcation into common iliac artery/vein was exposed and mobilized by ligating gonadal artery and vein, iliolumbar, lumbar branches and left adrenal vein using 6-0 silk sutures. The left ureter was identified and preserved with its arterial supply from the left renal artery. After mobilizing the abdominal aorta and IVC, 4-0 silk ties were placed around the aorta above the left renal artery, below the ligated iliolumbar branch, and at the bifurcation inferiorly. 500IU of low molecular weight heparin was injected through the penile vein. The distal aorta and IVC were ligated using the 4-0 silk suture and a vascular bulldog

clamp was placed distal to renal artery to occlude the aorta for cannulation.

In vitro Specific Absorption Rate (SAR) characterization of CPA+IONP cocktails

Synthesis and physical characterization of sIONPs and CPAs is described elsewhere [8,19]. For calorimetric estimation of SAR, the following methodology was adopted. The calorimeter consisted of an insulating sample holder placed within the solenoid induction coil. The magnetic field amplitude at 180 kHz was fixed in the range of 20-63 kA m^{-1} peak by changing the power supply voltage. The specific absorption rate (SAR) is defined as the measured thermal loss power normalized by mass of magnetic material (Fe for iron oxide IONPs) and expressed in units of W g^{-1} Fe calculated using the expression,

$$SAR = (C_p/m_{Fe}) \times (\Delta T/\Delta t) \qquad \text{Equation S1}$$

where T is temperature in °C, t is time in s, m_{Fe} is the equivalent mass of Fe in g, and C_p is the sample specific heat capacity in J g^{-1} $°C^{-1}$). For the SAR measurement, 1 g nanoparticle suspension in CPA at 10 mg Fe mL^{-1} was placed in standard 5 ml polystyrene tubes and inserted into an insulating sample holder. Fiber-optic temperature probes and an optical conditioner (Qualitrol T/Guard, Fairport, NY) were used to measure temperature in situ. The temperature probes were corrected for offset over the cryogenic temperature range (0 to -150 °C) by calibrating against a Pt100 RTD (Omega, Stamford, CT) in 20 °C intervals over the entire range, and performing a least-squares polynomial fit to the offset (R-squared 0.99, RMSE 0.50). Before commencing the measurements, each nanoparticle sample was allowed to equilibrate with the environment to ensure that the measured temperature was constant with a maximum deviation of ± 0.1 °C in 10 s. Temperatures were recorded every 1 s. The AMF power supply was turned on and temperature was monitored for 60 s or until maximum temperature of 60 °C, whichever was

achieved first. CPA blanks were used at each field setting to correct for calorimeter heat capacity by subtraction. The SAR values were evaluated for IONPs for 63 kA m^{-1} (peak) and at fixed frequency of 180 ± 5 kHz. For cryogenic evaluation, samples were first vitrified in LN2 vapor phase and stored at -150 ºC until deviation <± 0.1 °C in 10 s was observed. Then the samples were transferred over LN2 vapor to the calorimeter and power supply is turned ON instantly after placing the sample in the center of the coil. Convective heat transfer coefficient, h(T), is calculated using CPA control shams and a fitting function is obtained using least-squares fitting. Thus, SAR is calculated by subtracting convective heating effects from the thermometry data.

Perfusion circuit setup and kidney connection protocol

The basic schematic and layout for the perfusion circuit is shown in Figure 2A. For organ perfusion, we constructed a digital LabVIEW-controlled perfusion circuit, capable of performing hypothermic temperature-controlled step-loading of VS55 and IONPs while monitoring perfusion pressure in real-time. The following components of the perfusion circuit were purchased through Radnoti Inc (Covina, CA): 5.5 mL heating coil with integral classic bubble trap, bubble trap compliance chamber, preparatory custom tissue bath (80 mm x 20 mm), Tygon water-jacketed tubing, quick disconnect threaded caps, Q.D. plastic sleeve inserts, and 0.2 µm PES filters. A 4-channel, 8-roller digital peristaltic pump (Ismatec Reglo ICC) was purchased from Cole-Palmer (Vernon Hills, IL). Additionally, the following parts were purchased from Cole-Parmer: peroxide-cured silicone tubing (1.85 mm ID), silastic tubing (0.76 mm ID, DowCorning), 1/16" hose barb adapter, male luer with lock ring, Pendotec PressureMAT sensor with cable and luer fittings. A thermocouple probe with luer fitting was purchased from Biorep (Miami Lakes, FL). Labjack U6 DAQ was purchased from Labjack (Lakewood, CO). A

circulating chiller, Julabo F32 with HP controller (range, -20°C to 100°C) was purchased from Boston Laboratory Company (Woburn, MA). LabVIEW (National Instruments, Austin, TX) codes for controlling the pump speed and real-time pressure / temperature recording using the DAQ, were written in-house. Pressure / temperature data was recorded at a frequency of 1 data point/sec. Flow rates (mL min^{-1}) were experimentally calibrated vs pump rpm and validated against calculated values for a given tubing inner diameter. For the hypothermic perfusion setup, no oxygenator is used and there is no recirculation of CPA. The hypothermic perfusion parameters are listed in table S4. Temperature regulation of perfusate and organ chamber to set-point (e.g., 0°C for hypothermic), tubing connection, and circuit-priming are conducted before organ retrieval to minimize cold ischemic times (≤ 30 min). The shortest possible tubing length is used to maintain temperature and avoid kinks. For perfusion experiments the peristaltic pump is started, the circuit is primed, and the system is tested for leaks and air-bubbles while circulating buffer/carrier solution is flowing. Perfusate temperature is tested and perfusate flow rate is adjusted to low speed (< 1 mL min^{-1}) prior to kidney connection. For cleaning between experiments, all glass components are perfused and washed in a soap-bath and rinsed 3x with bleach + DI water, 3x DI water and 3x with ethanol.

For cold perfusion, the circulating chiller is set to -4°C and coolant circulation through the water-jacketed circuit components is turned on. 50 mL cold Euro Collins (EC) is poured into the perfusate reservoir maintained at 0-4°C on ice and circulation through the circuit is started. Leaks and air-bubbles are removed through tilting and tapping the lines. The program for pressure/temperature recording is started and a baseline pressure is acquired at 1.5 mL min^{-1}. Next, the EC flowrate is reduced to < 1 mL min^{-1} and the aorta of the kidney is connected to the perfusion circuit outlet tubing (Figure 2A) while keeping the cannula submerged in UW in the

cold-storage container to prevent air bubbles. The kidney is quickly and carefully transferred to the cold organ bath chamber while maintaining connection to the circuit. The vena cava is checked for effluent flow and is connected to a drainage tube for collecting effluents periodically (no recirculation), Figure S4.

Controlled Rate Freezer (CRF) and thermometry protocol for vitrification

CRF Program: The CRF is pre-programmed to start at a chamber temperature of T_c=0°C and cool down to T_c = -121°C at a ramp rate of -40°C min^{-1} (this rate was ascertained through previous *in vitro* thermometry tests on bags containing CPA only). A 25 min anneal step is introduced at -121°C (just above T_g) to allow the organ to equilibrate right before glass transition. A slower ramp rate of -10 °C min^{-1} was introduced to cool from -121°C to -150°C in the glassy phase to minimize thermal gradients. At T_c = -150 °C, a 30-minute temperature-hold step is programmed to allow the whole organ to equilibrate to the storage temperature (-150 °C) before transfer to a storage freezer at the same temperature.

Kidney Vitrification: After insertion of fiber-optic probes, the kidney is placed in a VS55+sIONP-filled cryobag and submerged in wet ice. The temperature probes are bundled and directed out of the bag through a narrow opening at the top of the bag. The kidney and the surrounding VS55+sIONP occupy an approximate volume of 5.5 cm x 4.5 cm x 1.5 cm. Excess air is evacuated from the bag, the bag is sealed, and temperature probes are affixed with transparent tape (3M, Minneapolis). The bag is closely wrapped with Al foil, pressing any air out between the foil and the bag. The foil-covered bag is next placed on a custom holder, which is introduced inside the CRF at T_c=0°C. Within the CRF, prior to starting the cooling program, the orientation of the bag/holder is adjusted so that the LN2 vapor flow is parallel to the flat bag faces, as shown in Figure S6. Temperature probes are directed out of the CRF through a vent and

connected to the multi-channel temperature data-logger (Qualitrol T/Guard, Fairport, NY). The data-logger is programmed to record temperature at a 1 s interval and is started. The CRF is sealed closed, following which the kidney is allowed to equilibrate to 0°C. Once a stable baseline is achieved at 0°C (5 min), the cooling CRF program is initiated. The total ischemic time between disconnecting the kidney from the hypothermic perfusion circuit to onset of cooling for vitrification was 17.5±2.5 mins (Table S5). After the temperature hold step at -150°C is reached, the kidney is allowed to equilibrate for at least 30 min, during which the temperature at different points in the kidney are monitored in real-time to ensure equilibration. At the end of the protocol, when the kidney has equilibrated to -150°C, the foil covered bag is quickly (2-3 s) transferred over LN2 vapor to a -150°C cryogenic storage freezer (MDF-C2156VANC-PA, Panasonic, IL). Here, the kidney is further stored for a total time of > 30 min (or theoretically indefinitely) to ensure equilibration, while the RF coil is prepared for nanowarming and the perfusion circuit for sIONP washout. The foil layer is peeled off from the bag, inside the storage freezer. Success or failure of vitrification of these kidneys was demonstrated by visual inspection and μCT analysis.

General considerations during cooling and rewarming

Successful vitrification is an optimization problem contingent on balancing cooling rates and thermal gradients. Therefore, the following general considerations are made in designing the cooling protocol:

(*i*) Cooling rates > CCR: The minimum cooling rate required to out-run ice crystallization from T_m to T_g (or more appropriately, homogeneous nucleation temperature T_h to T_g), termed as the Critical Cooling Rate (CCR), is a property of the CPA concentration and composition[7, 27]. For 8.4 M VS55, the CCR is -2.5ºC min^{-1} (Table S3). Therefore, the chamber cooling rate is adjusted

to -40ºC min^{-1} to allow rapid cooling through convection (rates determined through in vitro experiments in VS55 at volumes same as organ experiments).

(*ii*) Minimizing thermal gradients below glass transition: Adding an annealing step just above glass transition, which is long enough to allow temperatures across the kidney to equilibrate before entering the glassy state, will ensure minimal thermal gradient at the onset of cooling in glassy state. *Mehl et al* showed that, for VS55, ice nucleation occurs predominantly between -90ºC and -135ºC, whereas crystal growth is maximal above -85ºC[22]. Thus, in the design of cooling and rewarming protocols, annealing steps are limited to temperatures below -90ºC to reduce the probability of ice crystal growth. Additionally, a slow cooling rate (-10ºC min^{-1}) within the glassy state to the storage temperature (-150ºC) will prevent gradients, and thereby thermal stress, from building up as the kidney is further cooled to the storage temperature.

(*iii*) Organ container and geometry: A cryobag capable of withstanding temperatures to -150ºC was chosen as the container to vitrify the kidney because: (a) the thermal expansion coefficient matches VS55 more closely (compared to a tube, or a hard container)[16], thus reducing strain during cooling and rewarming; and (b) the shape flexibility of the bag allows use of smaller volumes of CPA for immersion of kidneys, and therefore enables more rapid cooling. Furthermore, the shape flexibility allows molding the geometry to a form that reduces stresses (aspect ratio ~ 1)[16] and allows the vitrified kidney and bag to fit the nanowarming RF coil. Additionally, an Al foil covering over the bag was used during cooling to cool the bag uniformly through conduction between the foil and bag, compensating for the asymmetry of forced convection resulting from the unidirectional flow of LN2 in the CRF (Figure S6). The foil also allowed molding the bag geometry to fit the RF coil.

As shown in Figure 3A (black dotted line) the chamber temperature profile meets all these performance requirements.

It is instructive to note that for T<-90ºC, nucleation of ice crystals is expected, especially during the 25 min annealing step at -121ºC, however ice crystal growth kinetics are too slow at these temperatures to have a to result in significant ice formation[22]. However, during rewarming, avoiding growth of ice crystals from pre-existing nuclei (formed during cooling), is a challenge, and therefore require rates of temperature change during rewarming that are even greater those during cooling in order to "outrun" ice growth. In Figure 4, perfusion of kidneys with VS55+sIONP (10 mg Fe mL^{-1}) and surrounding it with VS55+sIONP (4mg Fe mL^{-1} (n=3) or 10 mg Fe mL^{-1} (n=4)) ensures sufficient rewarming rates (> CWR) to avoid ice expansion and low gradients in the glass region to prevent fractures. However, 10 mg Fe mL^{-1} in the solution surrounding the kidney can cause the surface of the kidney to reach 0°C or higher, at which CPA toxicity is increased, while the medulla has not risen above melting (T_m). This can cause unintended toxicity on the surface of the kidney due to higher temperatures while exposed to CPA. Thus, while 10 and 4 mg Fe mL^{-1} were used in the thermometry cohort shown in Figure 4A, 4 mg Fe mL^{-1} was used in the suspension solution for all biological endpoints.

µCT setup for imaging vitrified kidneys

The vitrified kidney in the cryobag and was held at LN2 vapor temperature (-150°C) in a styrofoam container during imaging. Separate tubes of water and air at RT were attached to the top of the container to serve as calibration references for determining Hounsfield unit radiodensity. A typical scan takes 30 min to finish, and temperature history monitored in the sample chamber during that 30 min ranged from -174 to -158°C, well below the glass transition temperature. The images were reconstructed to reduce the beam hardening artifacts and improve

image quality (3D CT pro, Nikon Metrology, MI). The images were converted to unsigned 16-bit float images, post-processed (VGstudio Max 3.2, Volume Graphics, NC), and exported as DICOM images for a final analysis using MATLAB (MathWorks). The scale in HU was determined rom calibrated values based on the air and water sample densities, and the resulting images were used to document successful vitrification as frozen samples and vitrified ones have different density and appearance[44].

Computational heat transfer modeling

The computational geometry consists of kidney domain and bag domain containing the 100% VS55+sIONP (4 mg Fe mL^{-1}) solution. The bag is approximated to have a VS55+sIONP volume of 25 mL, modeled as 5.5 cm high, 4.5 cm wide and 1.5 cm thick with an ellipsoidal cross-section from top view and parabolic cross-section from side-view. The rat kidney is modeled as an ellipsoid with a major axis as 2 cm and the other two of its minor axes as 1 cm keeping the overall volume of kidney ~ 1 mL consistent with anatomical literature values[45 46].

$$q_v''' = SAR_{Fe}.C_i \qquad \text{Equation S2}$$

The thermal properties are chosen for the vitrified CPA (VS55) solution inside the bag and kidney assuming CPA equilibrated in the kidney tissue. In particular, k = 0.3 W mK^{-1}, C_p = f(T) (specific heat for VS55 is used as a function of temperature for the computational domain[12, 47] and ρ= 1100 kg m^{-3} [47]. Table S1 summarizes thermal properties.

The numerical solution to the non-homogenous transient heat transfer equation (Equation 1) is solved in the commercial FEA code COMSOL Multiphysics 5.5. The mesh was created using the finer option in COMSOL meshing code with tetrahedron elements consisting 5955 mesh vertices and 31226 tetrahedra. Convergence of solution was verified using COMSOL in-built physics-

controlled feature with relative error of 0.01%. Further, mesh convergence was evaluated using different mesh element sizes from the physics-controlled mesh submenu in the mesh node of study. The simulation is performed for 2 different cases during cooling i.e. cooling in CRF and LN2 plunge cooling and similarly 2 different cases during rewarming i.e. nanowarming and convective warming in water bath. The initial temperature of domain is at 4°C during cooling and -138°C during rewarming. At the boundary of computational domain i.e. bag, the heat transfer mode of interaction between the domain of interest with the surroundings is considered by only convection of LN2 vapors in the cooling chamber for case of convective cooling in CRF as well as LN2 plunge case with different heat transfer coefficients in both cases. In the rewarming cases, the heat flux across the bag during nanowarming case is approximated as natural convective boundary condition whereas forced convection of water (at 37°C) is the mode of heat transfer at the boundary in convective warming (WB) case. Detailed information regarding boundary conditions such as "h" values, T_∞ and additional details can be found in Table S6. Also, the details regarding the mesh size, number of elements, and convergence can be found in Figure S9. A simplified thermal shock equation Equation S3 was used to calculate the temperature difference corresponding to stress-to-fracture for VS55.

$$\sigma_T = g \times \left(\frac{E\beta\Delta T}{1-\nu}\right) \qquad \text{Equation S3}$$

where the Coefficient of Thermal expansion,

$$\beta = 1.91 \times 10^{-4} + 5.31 \times 10^{-7}T \qquad \text{Equation S4}$$

g is a geometric coefficient = 0.5 approximated for the modeled geometry, ν is Poisson's ratio = 0.2, E is the modulus of elasticity = 1 GPa and σ is the tensile strength of VS55 = 3.2 Mpa. After inserting all values, the limiting value (i.e. minimum) ΔT_{max} ~ 38°C. In Figure 5D, experimental

CRF cooling rate (black) was computed by taking the average of all temperature probes within a kidney, and then averaging this data over n=7 kidneys. For modeling, CRF CC *dT/dt* (blue) was computed by taking the average *dT/dt* of the maximum and minimum temperature limits across the modeled kidney volume. In Figure 5I, experimental NW (black) was computed by taking the average of all temperature probes within a kidney, and then averaging this data over n=7 kidneys. For modeling, NW *dT/dt* was computed by taking the average of the maximum and minimum temperature limits across the modeled kidney volume. Fe estimation in the medulla and cortex were obtained through calculations and analytical measurements- assuming average vascular volume fraction in entire rat kidney as 20% (20-25% is generally the vascular volume fraction in a kidney[45,46], calculated for limiting case 20%). cFe (total) = 0.2*10 mg Fe mL^{-1} * 1 mL = 2 mg Fe (vascular volume ~ 1mL). Alternately, based on ICP-OES[19], total Fe in kidney = 0.012 (mg Fe mg^{-1} dry wt.) * 163.7 (mg dry wt.) = 1.966 mg Fe. Using this information, and $(cFe)_{medulla}/(cFe)_{cortex}$ ~ 2 from MR imaging (Figure 2), $cFe_{medulla}$ ~ 2.15 mg Fe mL^{-1} (tissue concentration), cFe_{cortex} ~ 1.075 mg Fe mL^{-1}.

ICP-OES for Fe quantification in kidney washout samples

Each kidney sample was dried in a vacuum oven (LT1495X2, Thermo Fisher Scientific, Waltham, MA) and weighed. A dry weight of 40-50 mg was used for digestion. Each weighed tissue sample was then transferred to a 7-ml teflon microwave digestion vessel (Savillex Corporation, Eden Prairie, MN) to which 2 ml of concentrated HNO_3 (Fisher Scientific, Columbia, MD) was added. The vessel was sealed and placed into a 60-ml Teflon microwave digestion vessel (Savillex Corporation, Eden Prairie, MN) to which 10 ml of ultrapure H2O (Millipore Corporation, Billerica, MA) was added. Microwave digestion was performed in a household microwave as per the following protocol modified from[48] – 30 s heating at full power

and cool to ambient. After cooling, each sample was dissolved in 1:40 ratio in 10 ml of 0.5% HCl + 5% HNO3. Three reagent blanks were prepared the same way, except an acid-digest without the tissue was used to prepare final blank samples. External iron standards (TraceCERT®, Millipore Sigma, St.Louis, MO) at 100 PPB, 1PPM and 10PPM were used for calibration. Fe measurement and estimation was done using an iCap 7600 Duo ICP-OES Analyzer at the Research Analytical Laboratory at University of Minnesota.

MR imaging for IONP distribution

All kidneys were inserted into a 50 mL PET falcon tube and surrounded by the same solution that the kidney contained (EC or VS55) but without IONPs. The SWIFT 3D R1 map was acquired using a Look-Locker method with multi band (MB) SWIFT sequence for image readout using flip angle = 4°, acquisition delay ~ 2 μs, BW = 250 kHz, TR = 1.2 ms , gaps = 4, voxel resolution = 250 x 250 x 250 μm^3, and a total acquisition time around 13 min. The field of view (FOV) was 32 x 32 x 32 mm^3 with image matrix size = 128 x 128 x 128 x 24 (x,y,z,t), N_{spiral} = 64, N_v = 1024, and 64 time points spaced linearly from 12.7 to 1152 ms were acquired.

Diffusion time calculation for VS55 diffusion in tissue.

Diffusion time, $t \sim x^2/D$, where, x = mean intervascular distance in a kidney = 200 μm[45,46]. D = VS55 diffusivity in tissue = $7 \times 10^{-11} m^2 s^{-1}$ was obtained by μCT characterization of tissue-VS55 concentration vs time[37]. Therefore, diffusion time, t ~ 9.5 mins. This implies that, in theory, 15 min perfusion time at each concentration step should be sufficient for VS55 to diffuse into the tissue parenchyma.

Johnson-Avrami estimation of approximate ice formed during nanowarming

It is worth noting that in Figure 4A and Figure S7, there are regions in the temperature vs time

nanowarming plot, where the instantaneous warming rates drop slightly below the CWR (in the 40-50ºC min^{-1} range), especially in the cortex. Using the Johnson-Avrami model, originally developed for modeling ice crystallization kinetics during cryopreservation[22], the ice-fraction can be estimated from summation of the following equation:

$$X = \left(1 - e^{-[K(T).t]^n}\right) \quad \text{Equation S5}$$

, over the temperature range of -90ºC (*T1*) to -45ºC (*T2*), where *X* is the ice-fraction. According to *Mehl et al*[22], for VS55, an Arrhenius plot of the kinetic constant *k(T)* in a logarithmic scale vs the inverse of temperature generated a fitting function for *ln(k(T))* = 21.28-4661/*T*. In addition, an average estimate for the Avrami exponent over the temperature range of -90 to -45ºC was assumed based on experimental data in *Mehl et al*[22], n = 2.198. Next, a discretized form of the integral of Eq. S5 was used to estimate the ice-fraction. This analysis provides a rough approximation for the volume fraction of ice expected, assuming an Arrhenius dependence of nucleation and crystal growth rates on temperature. From this analysis, nanowarming results in an average ice volume fraction < 5%. In the worst-case scenario during nanowarming, where the rewarming rates are lowest in the cortex relative to other regions in the kidney, the estimated ice fraction in the cortical mass would be ~ 7%. While this ice fraction can be significant depending on the location and damage caused, the measured hypothermic pressures during sIONP washout after nanowarming show that the pressure was within acceptable range (<100 mmHg) in at least 2/3 of the kidneys (Figure S11) and responding to osmotic shifts similar to controls, suggesting minimal damage. Additionally, this ice fraction can be further reduced by increasing warming rates through one of the following means: (i) increasing volumetric concentration of sIONPs in the perfusate to c(Fe)>10 mg mL^{-1} (ii) using a nanoparticle with higher SAR under the available field conditions, (iii) increase the frequency or field magnitude of the applied RF field. In the

current study, we limited c(Fe) in the perfusate to 10 mg Fe mL^{-1} because higher colloidal instability is more likely at higher concentrations which could result in a poorer washout of the sIONPs from the kidneys. Thus, in the current study, 10 mg Fe mL^{-1} was chosen as the concentration to achieve sufficient rewarming rates without adversely affecting washout. However, the impact of higher sIONP concentrations can be studied further.

Washout characterization methods in stable vs unstable colloids

In contrast to other methods for measuring residual iron content following washout of sIONP from kidneys, specific observations of arterial pressure during perfusion can indicate failure from perfusion of unstable CPA+IONP colloids. Generally, colloid stability criteria are established prior to perfusion using *in vitro* methods – eg. an unstable colloid does not pass freely through a 1-4 µm PES filter. In addition, observation of arterial pressure during *ex vivo* perfusion can indicate instability. For eg, during kidney perfusion, a non-linear increase in perfusion pressure (P_{sIONP}) vs time or a high dP_{sIONP}/dt (>10 mm Hg min^{-1}) (Figures S3B and S14), could be a potential sign of blockages in the kidney vasculature from sIONP aggregates. High grade blockage can be tested by turning or pulsing the pump on and off while observing the pressure relaxation time to equilibrium. Blockage results in failure of the pressure to return to baseline – greater the blockage, longer the relaxation time to baseline following each pulse (gray shaded areas and blue dotted reference line in Figure S3B). Vascular occlusion from sIONP aggregates could result in localized high pressure and induce damage to the vasculature and endothelial lining as pressure exceeds physiological pressure limits, as evidenced in Figure S3I-K, S3N-U. Indeed, in the few cases where high washout pressures (>>100 mm Hg) were observed during perfusion with an unstable colloid, kidney characteristics post-washout included visual and histological confirmation of sIONP retention in the glomeruli and vasa-recta (Figure S3L-M) and

significant damage to the endothelium in blood vessels, especially in the medulla (Figure S3T-U). Additional characterization of a kidney using μCT, following sIONP loading at dP_{sIONP}/dt > 100 mm Hg min^{-1} (Figures S3I-K and S14), showed features consistent with sIONP aggregation in the kidney (compare to Figure 2K). High intensity pin-point spots in the cortex corresponded to IONP localization in the cortex, very likely in the glomeruli. Additionally, high contrast lines were observed in the medulla where medullary rays (collecting ducts and straight tubules) and the vasa-recta were present. Prussian blue staining indicated iron retention in the glomeruli in the cortex and inter-tubular space in the medulla. This likely indicated damage of the capillaries in the vasa recta and leakage into the inter-tubular space, appearing to take the geometric pattern of the medullary rays. In some cases, a yellow colored residue in the glomeruli and vasa recta were observed post-washout (Figure S5C), which stained negative for Congo Red and PAS, indicating very likely remnants of unstable sIONPs or coating left behind due to incomplete washout. Additionally, no sIONPs were observed in the effluent from the ureter (clear), suggesting that the sIONPs are less likely to enter the tubules and the collecting duct. This was further verified by Prussian blue staining of the kidneys, where no blue stains for Fe were observed in the tubules or collecting ducts (Figure S5B).

Characterization of sIONP size and magnetic properties before and after washout

Dynamic Light Scattering (DLS, Brookhaven Zeta PALS instrument, Holtsville, NY) was used to measure the mean hydrodynamic diameter of sIONPs in VS55 before loading and after washout. 400x diluted samples of 10 mg Fe/ml in VS55 from three separate kidney perfusion experiments were used for the measurement. Histogram depicting intensity-weighted contributions of size ranges (G(d)) and the cumulative percentage contributions (C(d)) were plotted vs size (d) (Figure S12).

Vibrating Sample Magnetometry (MicroMag VSM, Lake Shore, Princeton, NJ) was used to measure the DC hysteresis properties of sIONPs. Samples from kidney perfusion experiments were measured at room temperature to determine if they retained their superparamagnetic properties (Figure S13). A liquid helium cryostat allowed measurements of sIONP in VS55 at cryogenic temperatures. A stepper motor that rotates the vibration head was used to center the sample. Vibration amplitude of 0.2 and averaging time of 0.1s were used.

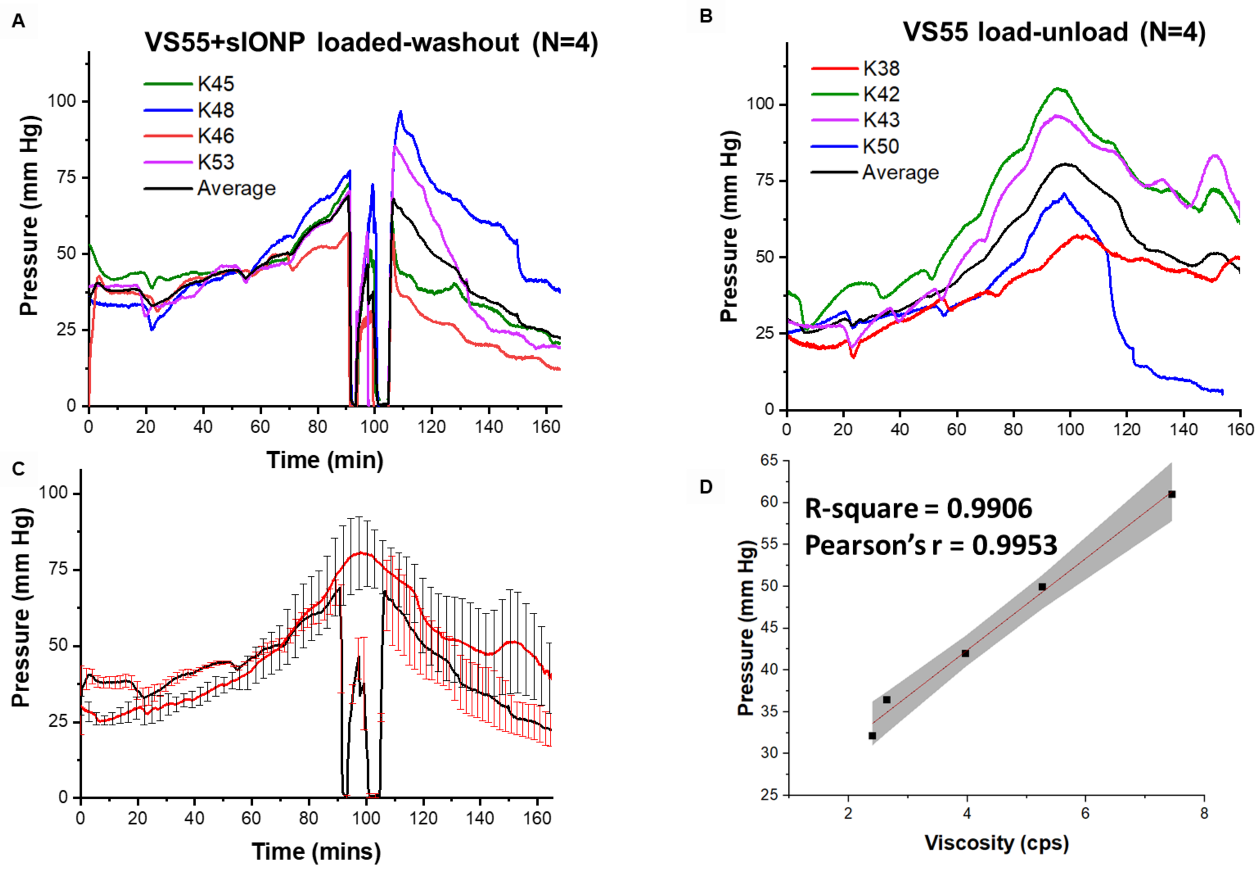

Figure S1. Pressure measurements during renal perfusion loading of VS55 only or VS55+sIONP. A) Arterial perfusion pressures in each of four different kidneys (green, blue, red, purple) and the average of all (black) during hypothermic perfusion loading and washout of VS55+sIONPs under constant flow conditions and step-loading. B) Arterial pressure in four different kidneys and their average pressure (black), during hypothermic perfusion loading and washout of VS55-only under constant flow conditions and step-loading. C) Overlay of arterial pressures during VS55+sIONP loading-washout (black solid line with red SEM error bars) with VS55-only loading-washout (red solid line with black SEM error bars). D) Linear regression of mean arterial pressure (from C) during each loading step vs mean perfusate viscosity (n=3) (red line), plotted with 95% CI interval (gray shaded).

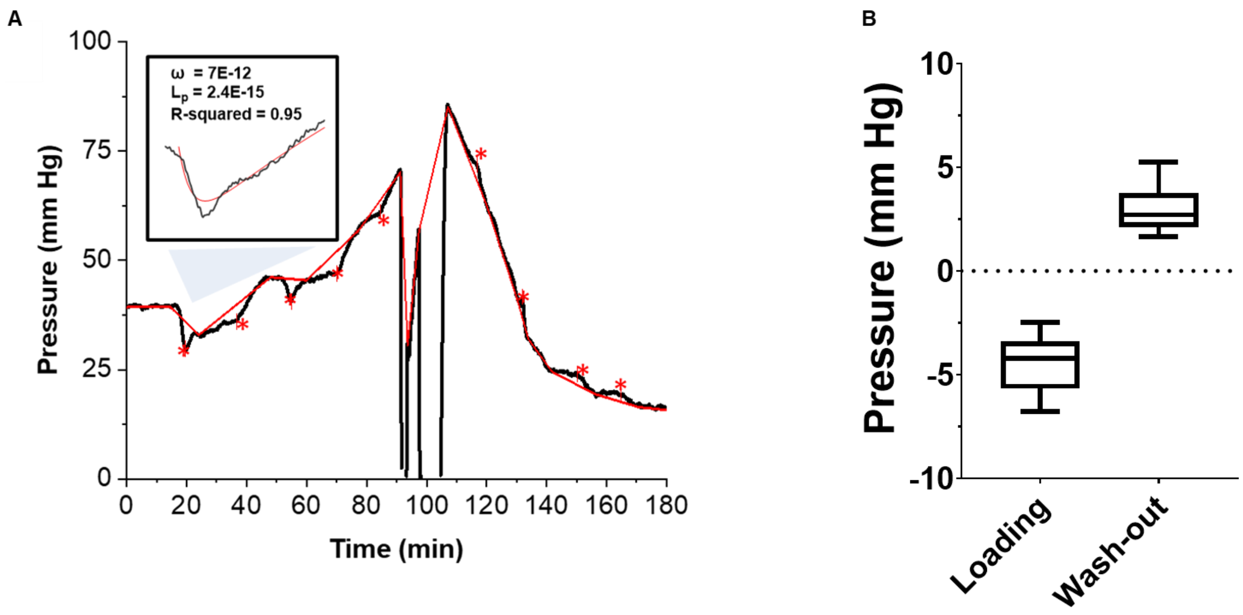

Figure S2. Pressure variation due to VS55 step concentration changes during hypothermic perfusion for VS55+sIONP loading and unloading. A) Representative plot of arterial pressure variation during step-loading and wash out of VS55+sIONP during a perfusion experiment. Red line in the main plot indicates a piecewise linear fit to the plot to determine peak height from baseline. Inset shows enlarged image of one peak arising from a shrink-swell event during loading with Krogh Fit (red line-inset) and fitting parameters (ω= $7*10^{-12}$ mol N^{-1} s^{-1}, L_p =$2.4*10^{-15} m^3$ N^{-1} s^{-1}). The multiple shrink-swell (loading) and swell-shrink (wash out) peaks where pressures typically drop or rise above the average are designated with an *. B) Box-whisker plot showing the peak-height distribution in n = 4 kidneys during loading and wash-out, arising from the shrink-swell (loading) and swell-shrink (washout) behavior of the artery lumen and tissue parenchyma in response to the osmotic gradient introduced in each loading and wash out step.

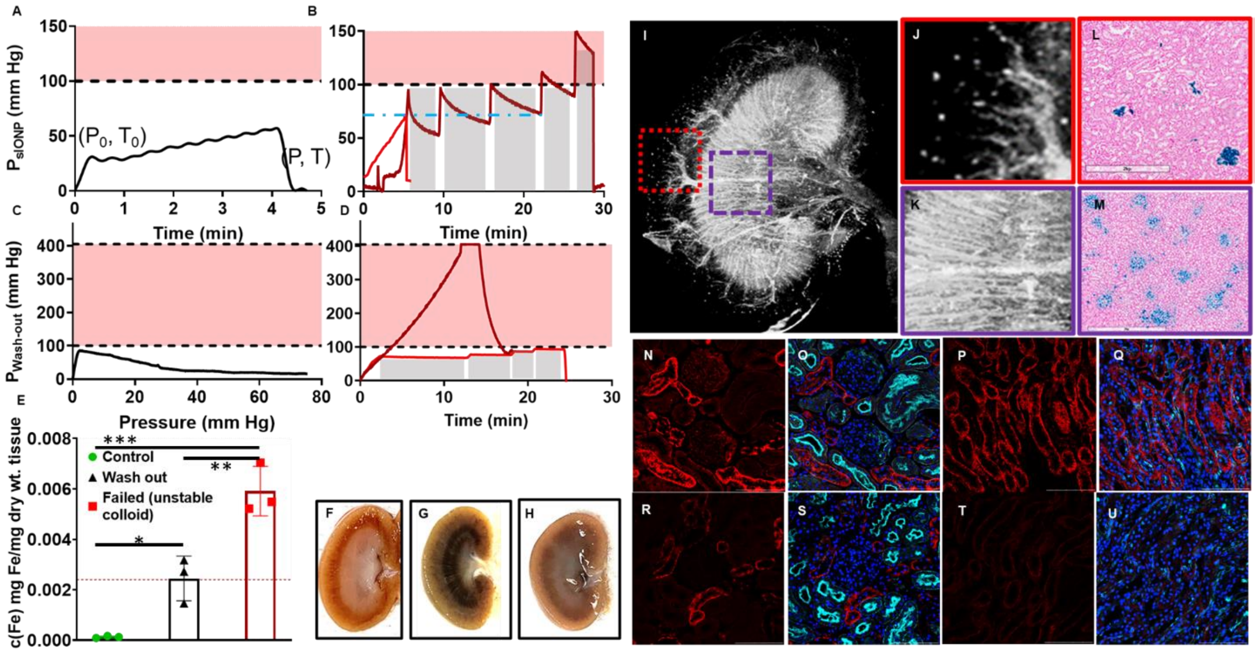

Figure S3. Characterization of successful vs failed kidney washout of different VS55+sIONP solutions (colloidally stable vs unstable). A, B) Kidney arterial pressure variation during sIONP loading, P_{sIONP}, for a stable colloid (passes through a 4 µm polyethersulfone (PES) filter = stable) and unstable VS55+sIONP colloid (clogs in a 4 µm PES filter = unstable), respectively. These different formulations resulted from studies of sIONP purification methods that resulted in alterations of stability in solution. The 0-100 mm Hg range (unshaded portions), demarcated by a dotted line at 100 mm Hg, overlaps with physiological arterial perfusion pressure for rat kidneys. The red shaded region indicates an undesirable pressure range and transgresses physiological limits (P>120 mm Hg). Gray shaded regions indicate temporal regions during the experiment, where the pump was switched OFF, to allow the pressure to relax back towards an unperturbed equilibrium state before turning it back ON. For stable colloids (A), low rates of rise of pressure (<10 mm Hg min^{-1}) are observed. For unstable colloids (B), high rates of rise of pressures (>10 mm Hg min^{-1}) are suggestive of aggregation and occlusions in blood vessels (Figure S14). This is evidenced by the increase in relaxation time to equilibrium (light blue dot-dash line),

following each pump ON-OFF sequence and a failed washout as shown in (D). C) Washout pressure, P_{wash}, from perfusion and washout of a stable VS55+sIONP colloid, where $P_{wash} < 100$ mm Hg through the washout and pressure decreases with time as the CPA concentration is reduced to baseline (buffer). D) Failed washout from an unstable VS55+sIONP colloid, where either (i) P_{wash} increases continuously, beyond the pressure sensor saturation (~400 mm Hg) under constant flow conditions (~0.5 ml min^{-1}) and physically ruptures the kidney (data not shown) or, (ii) P_{wash} does not relax to baseline pressure when pump is switched OFF to maintain $P_{wash}<100$ mm Hg, indicating the venous end (at atmospheric pressure) is blocked off from the arterial end. E) ICP-OES measurements (n=3 per group) comparing iron retention in mg Fe mg^{-1} dry weight for successful (stable colloid) vs failed (unstable colloid) washed out kidneys. 2.5-3x higher retention is observed in kidneys blocked from an unstable colloid. *$p<0.05$, **$p<0.005$, ***$p<0.0005$ (One-way ANOVA with Tukey's posthoc multiple comparisons test). For reference, LOD in the measurement is 1 ng mg^{-1}. It was previously shown that a kidney fully perfused with sIONP in VS55 (10 mg Fe mL^{-1}) displayed a c(Fe) ~ 0.012 mg Fe mg^{-1} dry weight[19]. Thus, in washed out kidneys, 20% or lower Fe is retained, whereas failed washout in kidneys (unstable colloid) corresponded to approximately 2.5x higher Fe than washed out kidneys (stable colloid). The red dotted line indicates a concentration of Fe previously observed (0.0024 mg Fe mg^{-1} dry weight)[26] in kidneys of mice which were *i.v.* injected with IONPs (0.18 mg Fe g^{-1} mouse), 24 hours post injection. This concentration appeared to be well tolerated by the mouse for at least a month. The ICP-OES data is generally in agreement with observation of gross hemi-sections of the kidney where (G) shows kidneys with a darker contrast in the medullary region after perfusion and wash out from an unstable VS55+sIONP colloid, compared to a washed-out kidney (stable colloid) (H). I) μCT image of a kidney perfusion loaded with

VS55+sIONP (unstable colloid) at a rate, such that rate of increase in P_{sIONP} > 100 mm Hg min^{-1}. J, K) show zoomed in pictures of the red and purple squares in (I), respectively. (J) shows X-ray contrast from Fe within the cortical glomeruli and (K) shows sIONP retention in the vasa-recta and leaked out into the extra-tubular space (along the medullary rays). L, M) Prussian Blue staining in kidney sections corresponding to (J) and (K) confirm Fe retention in the glomeruli and vasa recta. N, O) show CD31 and merge (Gal+DAPI+CD31) confocal microscopy images, respectively, in the cortex of a successful washed-out kidney (stable colloid). P, Q) show similar images for the medulla of a washed-out kidney (stable colloid). R, S) show CD31 and merge (Gal+DAPI+CD31) confocal microscopy images, respectively, in the cortex of failed washed out kidney (unstable colloid). T, U) show similar images for the medulla of a failed washout kidney (unstable colloid). Significant damage to the endothelium is observed in the case where washout failed following perfusion of an unstable colloid.

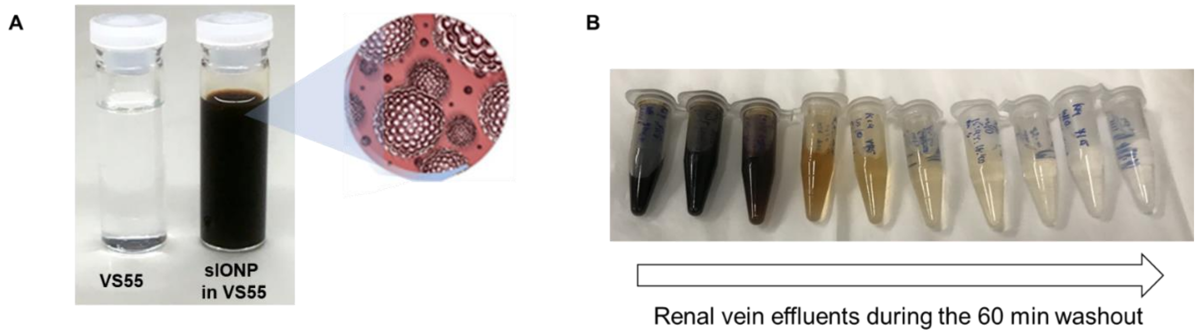

Figure S4. Gross appearance of VS55+sIONP colloid (stable). A) Glass vials containing 8.4 M VS55 (clear) and VS55+sIONP (8.4M and cFe=10 mg Fe mL^{-1}) (dark brown colloid). B) Renal vein effluents collected during the 60 min washout of the VS55+sIONPs (stable colloid) from the kidney.

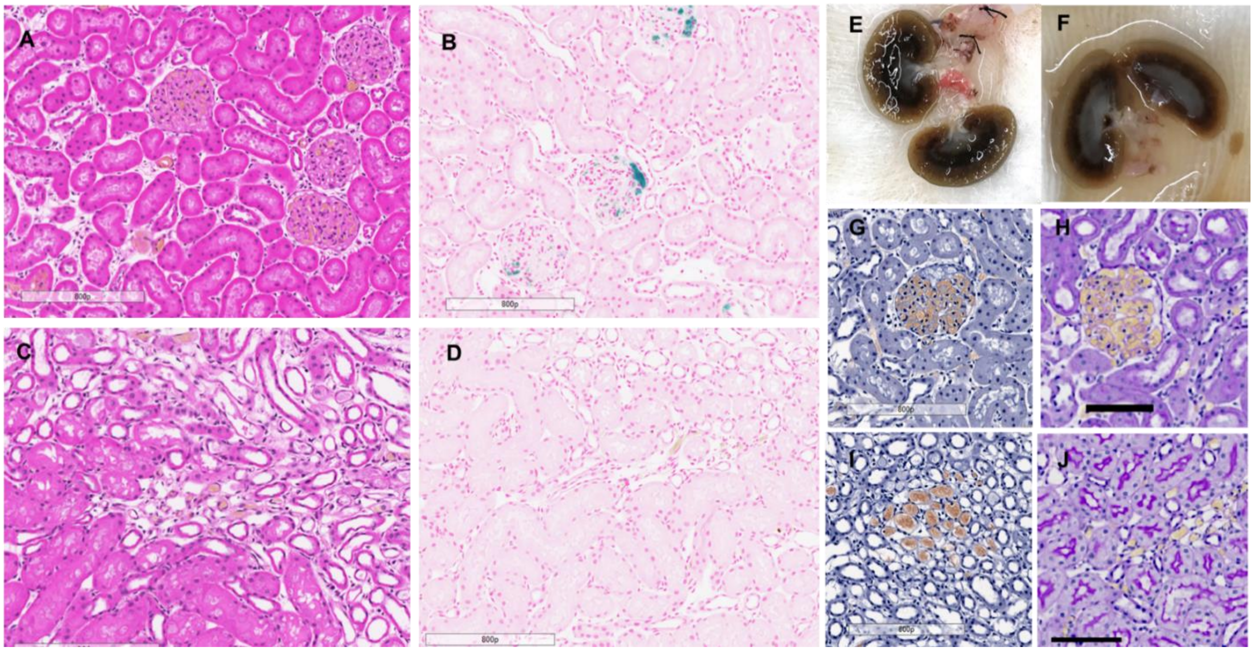

Figure S5. Histological characterization of kidneys perfused with a colloidally unstable VS55+sIONP solution. A, C) H&E images of cortical and medullary sections, respectively, of a kidney post-washout after perfusion of an unstable VS55+sIONP colloid formulation. The yellowish deposition in the glomeruli and some regions in the medulla stained negative for Congo Red (G, I) and PAS (H, J), indicating that the residue is unlikely a protein and more likely remnants from an unstable VS55+sIONP colloid batch. B, D) Prussian blue stained sections of the cortex and medulla, respectively, of the same kidney post-washout shows some Fe retention in the glomeruli which corresponds with the observed yellow coloration. E, F) Gross hemi-sections of similar kidneys post-washout (unstable colloid) show significant dark brown coloration in the medulla and some dark spots in the cortex, corresponding to sIONP retention. Scale bar = 200 μm. The unstable sIONP batches were attributed to an incomplete purification after synthesis.

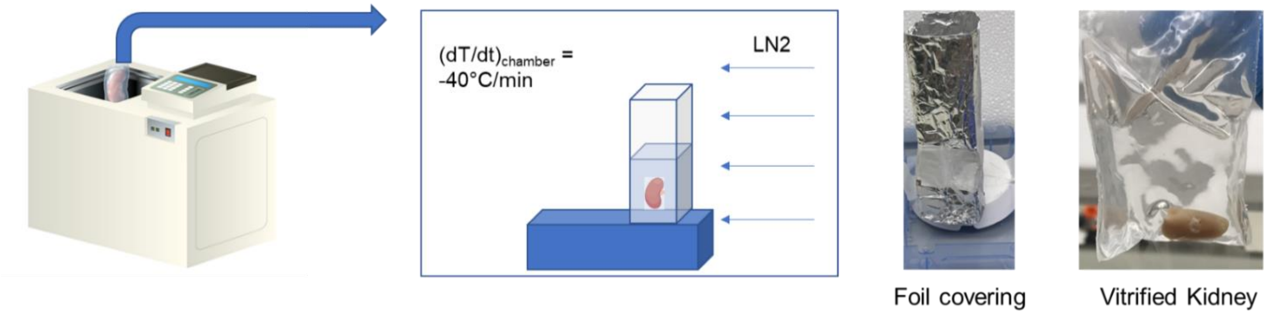

Figure S6. Controlled rate freezer (CRF) and container set-up for kidney vitrification. See supplementary methods (CRF protocol) for details.

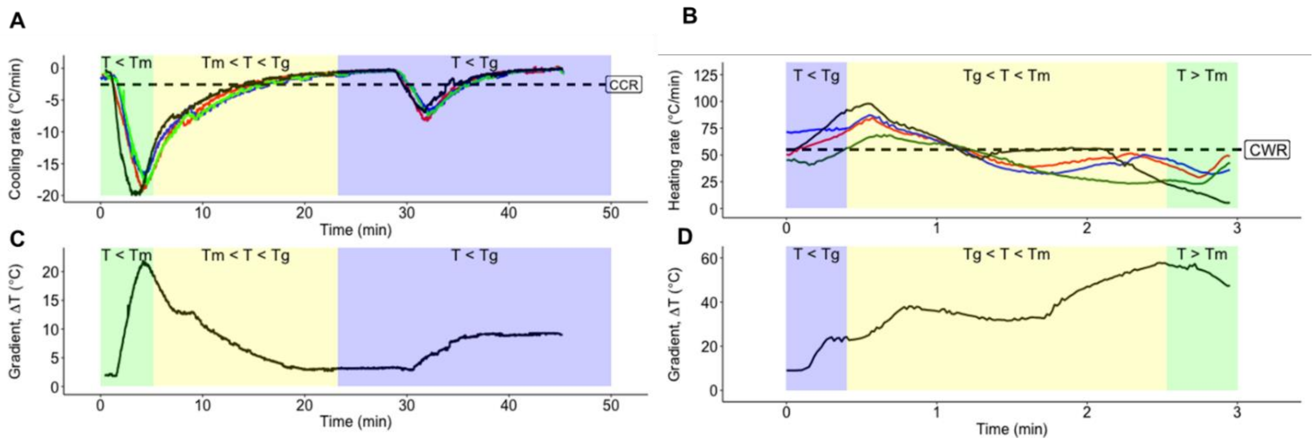

Figure S7. Cooling/rewarming rates and gradients vs time for vitrified and nanowarmed kidneys shown in Figure 3A and 4A, respectively. A, B) Cooling and rewarming rates vs time, corresponding to Figure 3A and 4A, respectively. C, D) Maximum gradient observed between the leading and lagging temperature probes, vs time during cooling and rewarming processes corresponding to Figure 3A and 4A respectively.

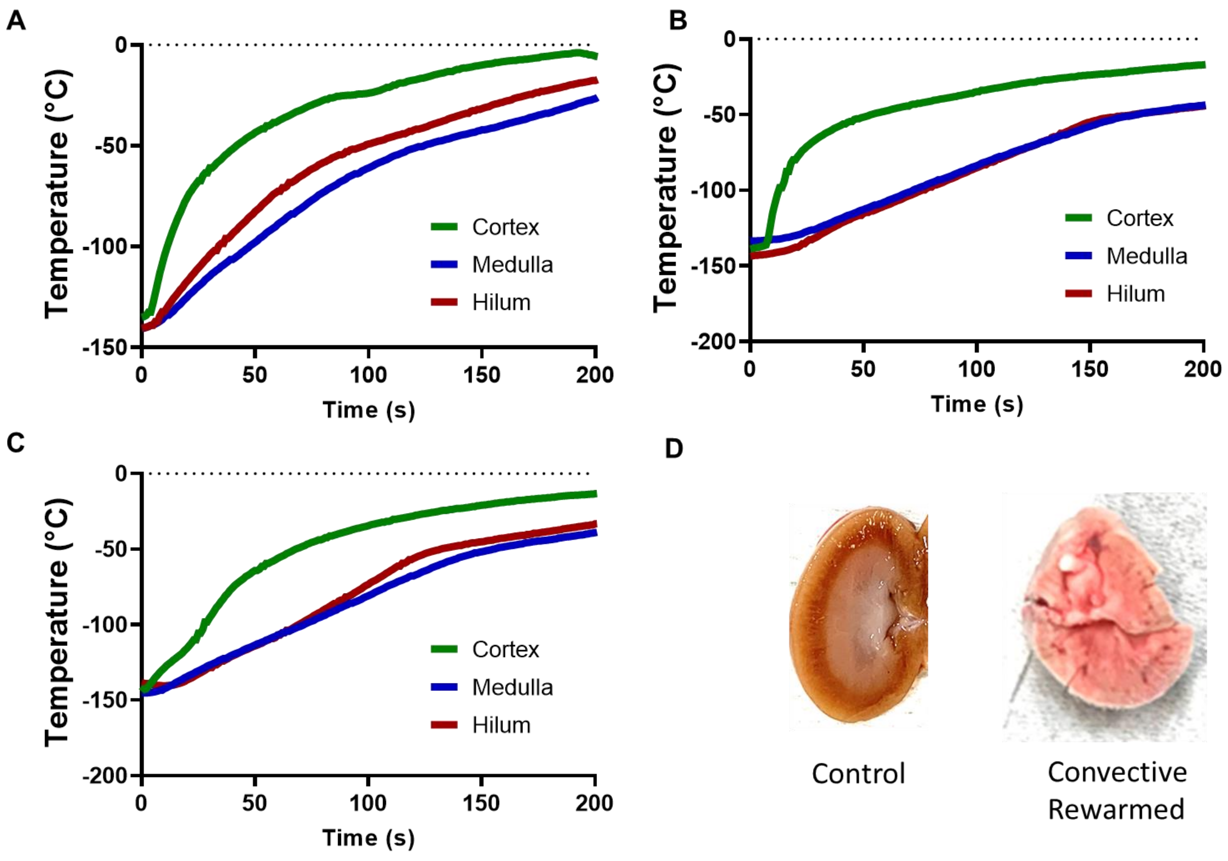

Figure S8. Thermometry for convectively rewarmed kidneys. A-C) Temperature vs time thermometry data for rewarming of vitrified kidneys using water bath (37ºC), measured at the cortex, medulla and hilum. Note the large temperature differences, especially between the cortex and the medulla during rewarming, which are greater than 38°C (stress-to-fracture gradient for VS55- Figure 5 and computational modeling section). Such large gradients can result in cracking and physically damage the kidney. Additionally, slower warming rates at the center of the kidney, where heating rates are diffusionally limited, ice growth can result in significant damage. D) Gross morphology in a hemi-section of a convectively rewarmed kidney (right) imaged immediately post rewarming when cortical temperature probe>T_m+10°C (~-28 to -35°C) with visible crystals (i.e. failure), in comparison to a control fresh harvested kidney.

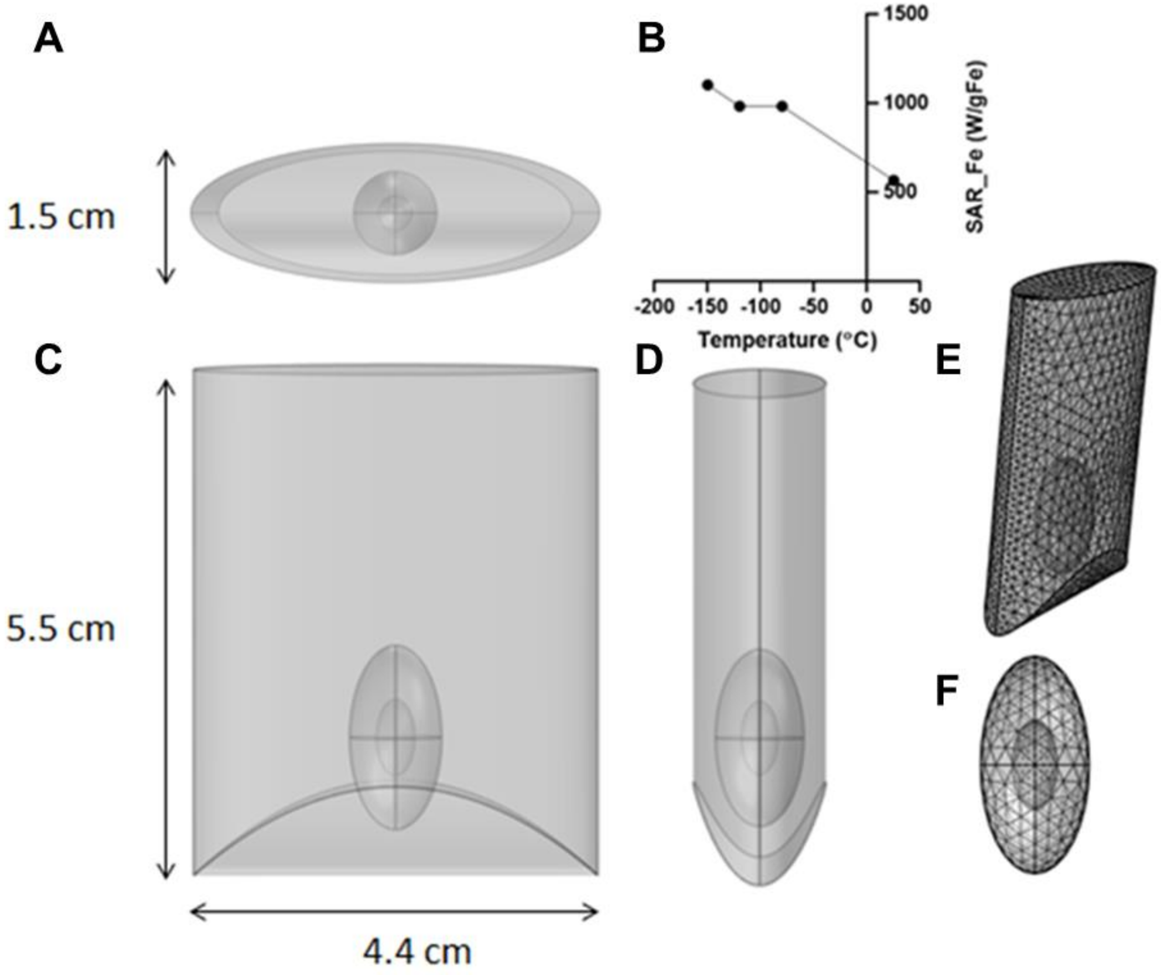

Figure S9. Kidney and container geometry for computational thermal modeling. A, C, D) Computational geometry of bag and kidney for the FEM analysis. E, F) represents the cross-section of bag and kidney domain showing the FEM mesh. The mesh is created using the finer option in COMSOL meshing code with tetrahedron elements consisting 5955 mesh vertices and 31226 tetrahedra. Convergence of solution was verified using COMSOL physics-controlled feature with relative error of 0.01%. Further, mesh convergence was evaluated using different mesh element sizes from physics-controlled mesh submenu in the mesh node of study. B) shows

the temperature dependent SAR approximation for sIONP used in the modeling.

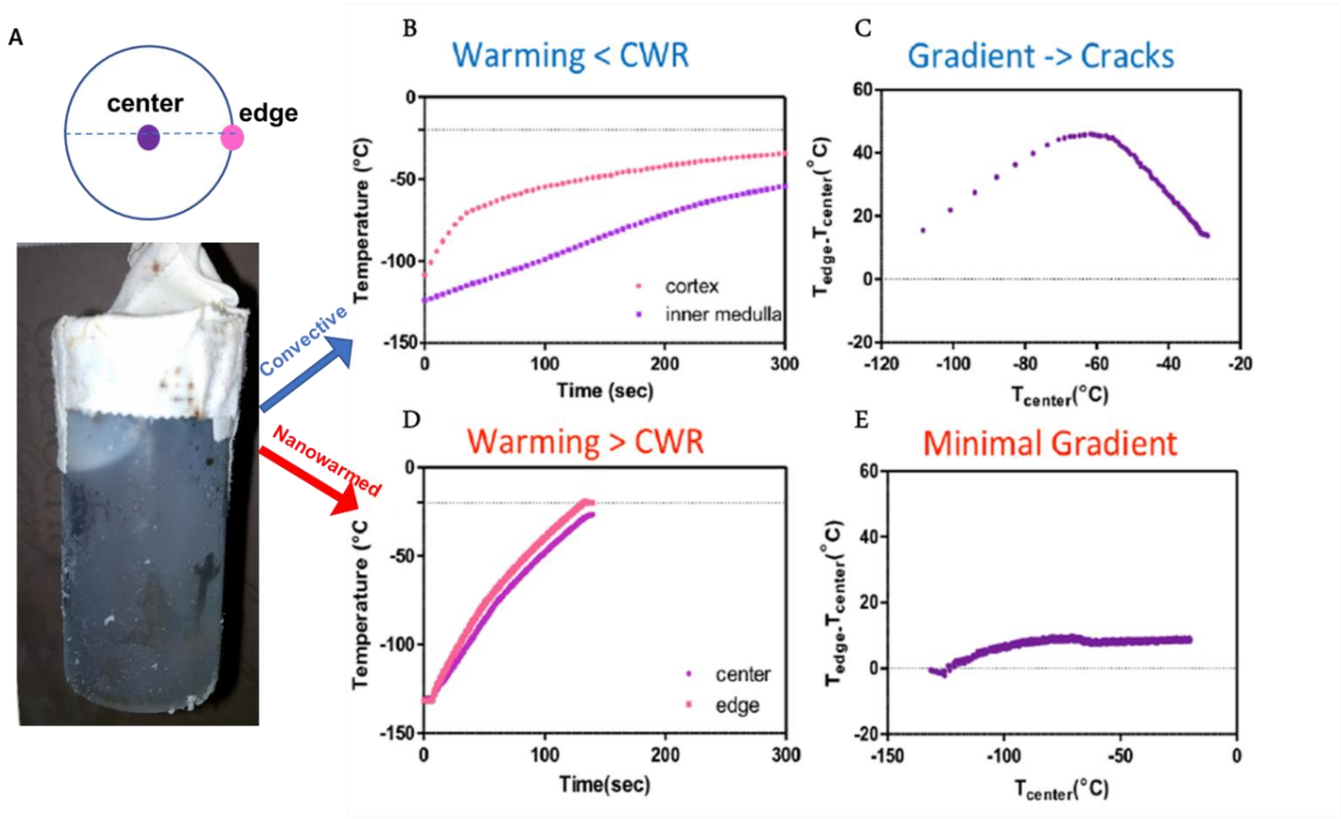

Figure S10. Thermometry during nanowarming vs convective rewarming of a vitrified rabbit kidney in M22. A) Gross Image of a vitrified kidney inside a tube containing M22+EMG308 IONPs, with schematic showing top cross-section and relative placement of fiber optic temperature probes at the center (inner medulla) and edge (cortex) of the kidney. B) Temperature vs time measured in a rabbit kidney at the cortex (pink) and inner medulla (purple) during water-bath rewarming. Data reproduced from *Fahy et al*[23]. Note that rewarming rates are greater than CWR for M22, but less than CWR for VS55. C) Temperature difference, ($T_{edge} - T_{center}$), between the kidney cortex (T_{edge}) and inner medulla (T_{center}), vs T_{center}, is a measure of the gradients during rewarming. If we approximate the stress-to-fracture temperature difference for M22 to be approximately same as VS55 (38°C), the gradient approaches this limit during water-bath rewarming for VS55. Data reproduced from *Fahy et al*[23]. D) Temperature vs time measured at the cortex (pink) and inner medulla (purple) during nanowarming. Note that rewarming rates are

greater than CWR for both, M22 and VS55. E) Temperature difference, ($T_{edge} - T_{center}$), between the kidney cortex (T_{edge}) and inner medulla (T_{center}) vs T_{center}, is a measure of gradients during nanowarming (much lower, ≤10°C, than the stress-to-fracture threshold for VS55, 38ºC).

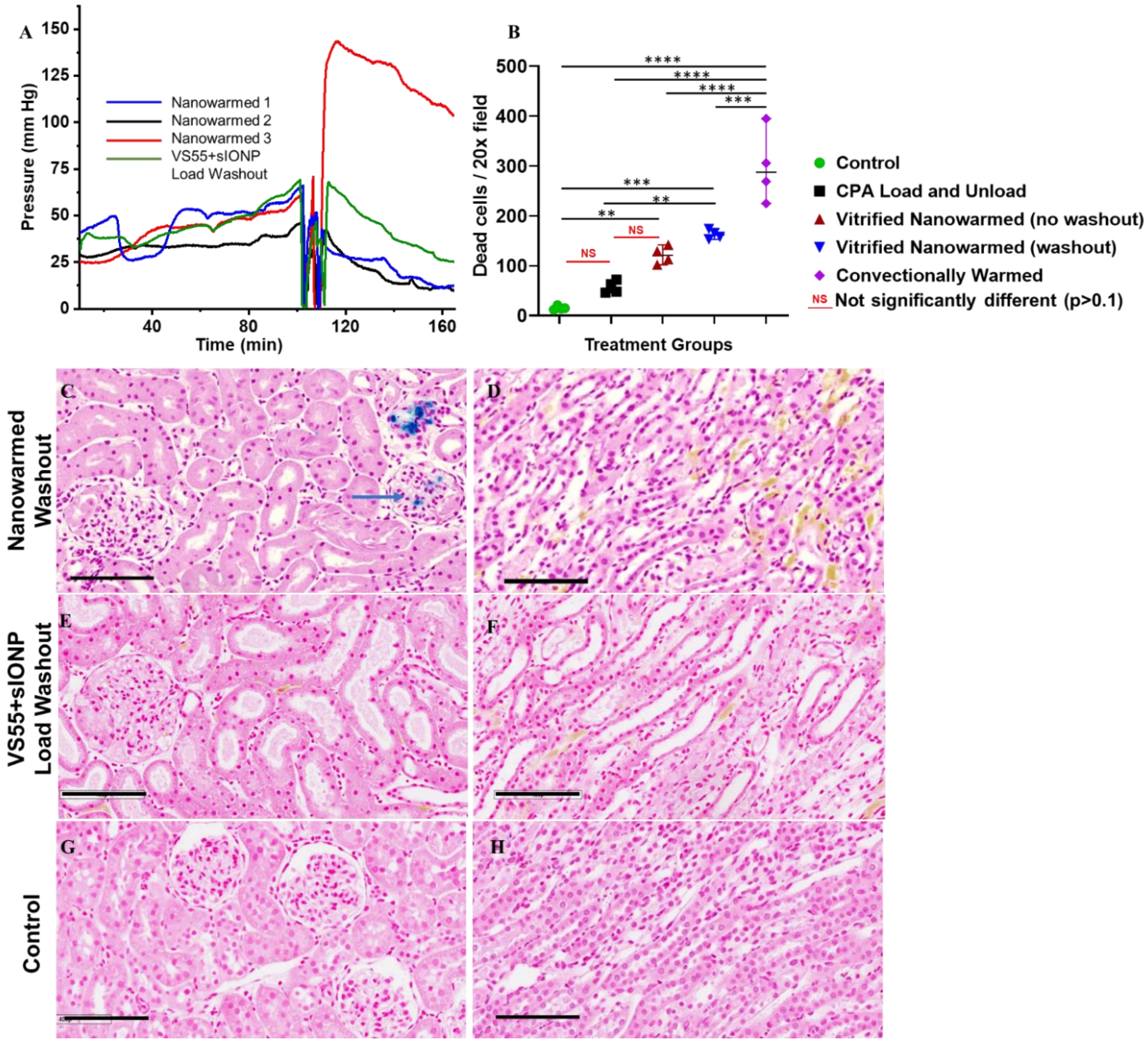

Figure S11. Perfusion pressure, viability (AO/PI) and histological characterization of nanowarmed kidneys and washed out vs VS55+sIONP loaded and washed out vs Control. A) Arterial pressures during VS55+sIONP loading prior to vitrification and nanowarming, and washout of VS55+sIONP from nanowarmed kidneys (starting at ~ 110 min) compared to mean pressure from VS55+sIONP loaded and washout kidney (green). From the three nanowarmed kidneys, one outlier is observed in washout responses under identical constant flow conditions (1.5 mL min^{-1}). Nanowarmed kidneys 1 and 2, show pressures within physiological range upon

washout, albeit lower than corresponding loading pressures at same concentrations of VS55. The superimposed swell-shrink pattern lies on top of the generally decreasing pressure curve during washout, suggesting the vasculature is responding to osmotic shifts. The lower washout pressure compared to controls could suggest shunting or leakage indicating some vascular damage. Nanowarmed kidney 3, however, shows higher wash out pressures. This could be due to nanoparticle aggregation in the vasculature, as suggested by the higher rate of rise of dP_{sIONP}/dt (>10 mm Hg min^{-1}) under same flow rates as other kidneys (0.5 ml min^{-1}). B) Scatter plot with median and range showing dead cell count per 20x field from AO/PI stained slices obtained from nanowarmed kidneys compared to controls. *$p<0.05$, **$p<0.005$, ***$p<0.0005$ (One-way ANOVA with Tukey's posthoc multiple comparisons test) for n = 4. No significant differences (red line, $p>0.1$) are observed between (i) nanowarmed kidneys (no washout ▲) and CPA loaded and washed out kidneys (■) and (ii) CPA loaded and washed out kidneys (■) and controls (●). C) Prussian Blue staining of a representative nanowarmed kidney section showing glomeruli in cortical region of the kidney. Some occasional blue spots (blue arrow) corresponding to remnant Fe were observed in a few glomeruli. D) Prussian Blue staining of a nanowarmed kidney section showing the medulla indicating no evidence of retained Fe. Similar to Figure S5, a yellow deposition was observed which is maybe related to the sIONP formulation. E) Prussian Blue staining of a VS55+sIONP loaded and washed out kidney section showing glomeruli in cortical region of the kidney. Like Figure 2(O), the glomeruli showed no evidence of Fe retention. F) Prussian Blue staining of a VS55+sIONP loaded and washed out kidney section showing the medulla indicating no evidence of retained Fe. Similar to Figure S5 again, a yellow deposition was observed which is maybe related to the sIONP formulation. G, H) Prussian Blue staining of a control kidney section showing the cortex and medulla, respectively. Scale bars indicates 100

µm.

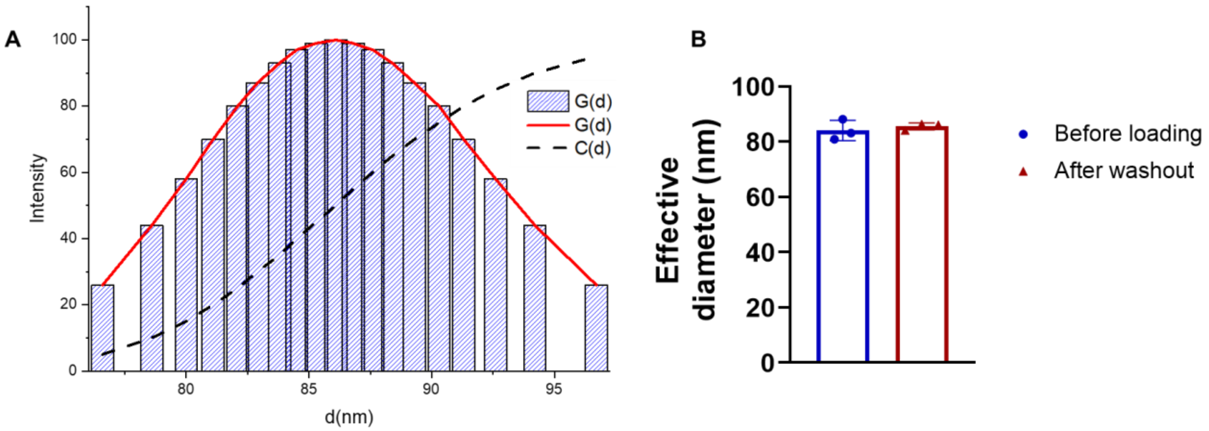

Sample	Before loading			After washout		
	Effective dia. (nm)	Std. Error	pdi	Effective dia. (nm)	Std. Error	pdi
K10	83.1	0.6	0.085	84.1	0.9	0.069
K14	80.9	0.2	0.047	86.4	1.1	0.028
K23	88.1	0.7	0.078	86.1	1.5	0.106

Figure S12. (A) Dynamic Light Scattering (DLS) data collected on CPA+sIONP effluent sample from a kidney, showing the intensity-weighted histogram plot G(d) for the distribution of sIONP hydrodynamic diameter. C(d) indicated the cumulative distribution. (B) Bar + Scatter plot comparing the mean hydrodynamic diameter of sIONPs before loading and after washout from three kidney perfusion experiments. Error bars indicate standard deviation for samples obtained from n = 3 kidneys. (c) Table summarizing effective diameters, standard error and polydispersity index (pdi) in sIONP loading and washout samples from 3 kidneys.

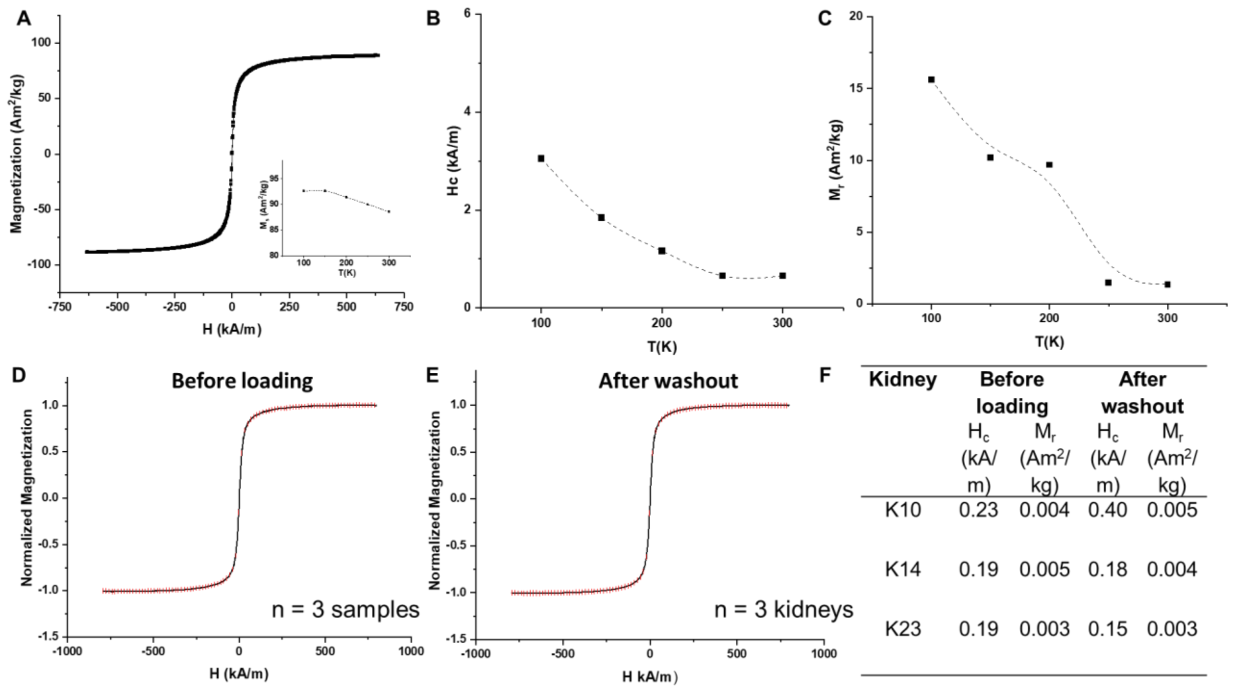

F

Kidney	Before loading		After washout	
	H_c (kA/m)	M_r (Am²/kg)	H_c (kA/m)	M_r (Am²/kg)
K10	0.23	0.004	0.40	0.005
K14	0.19	0.005	0.18	0.004
K23	0.19	0.003	0.15	0.003

Figure S13. (A) Vibrating Sample Magnetometry (VSM) measurements on a sIONP+VS55 sample showing DC hysteresis loops over a field range of +/- 625 kA/m at room temperature for sIONPs. The shape of the hysteresis is typical of superparamagnetic nanoparticles which exhibit no coercivity or remanence (loop area zero). The inset shows saturation magnetization measured over the cryogenic temperature range. (B&C) Temperature dependence of coercivity (H_c) and remanence (M_r), respectively, measured over the cryogenic temperature range. (D) DC hysteresis of sIONP samples in VS55 before perfusion loading of kidneys (n=3, error bars indicate standard deviation skipping every 10 points i.e. 1 s) measured between +/- 795 kA/m (E) DC hysteresis of sIONP samples in VS55 after washout from kidneys (n=3, error bars indicate standard deviation skipping every 10 points i.e., 1 s) measured between +/- 795 kA/m. (F) Table summarizing differences in coercivity (H_c) and remanence (M_r) characteristics between sIONP loaded and washout samples. H_c and M_r are both close to zero for superparamagnetic nanoparticles, which is consistently observed in both sets of samples implying no aggregation or change in magnetic

properties.

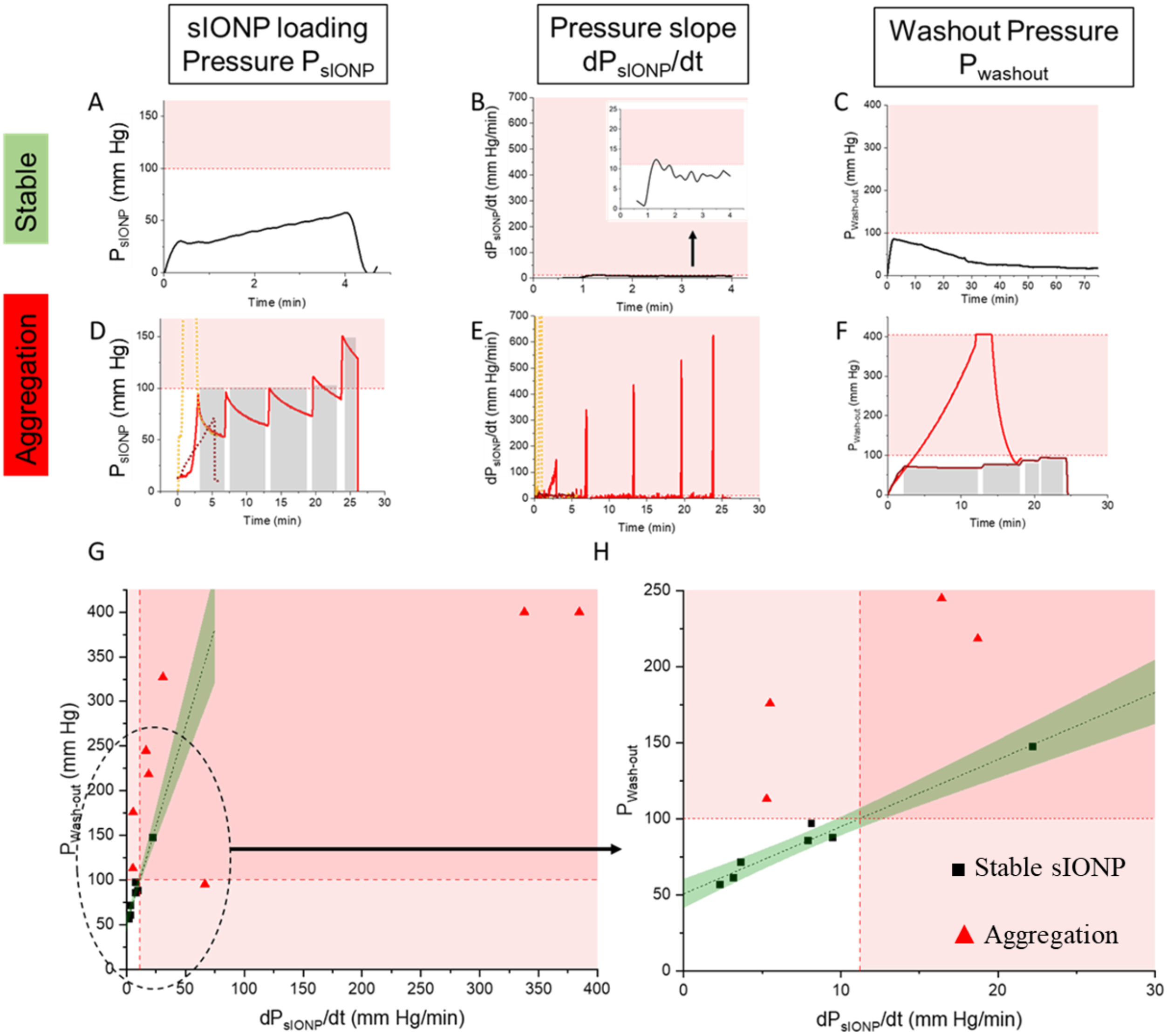

Figure S14. (A-C) sIONP loading pressure (P_{sIONP}), rate of increase of sIONP loading pressure (dP_{sIONP}/dt) and initial washout pressure ($P_{wash\text{-}out}$), respectively for a kidney optimally loaded with a stable sIONP+VS55 colloidal system. (D-F) sIONP loading pressure (P_{sIONP}), rate of increase of sIONP loading pressure (dP_{sIONP}/dt) and initial washout pressure ($P_{wash\text{-}out}$), respectively for a kidney loaded with an unstable aggregation-prone IONP+VS55 colloid (clogs a 4 µm PES filter). Failure cases (D-F) are characterized by high P_{sIONP} (>100 mm Hg), high

dP$_{sIONP}$/dt (>>10 mm Hg/min), and high initial washout pressures (>100 mmHg). (G) Correlation plot of $P_{wash\text{-}out}$ vs dP$_{sIONP}$/dt for stable (black squares) and unstable colloids (red triangles). Green-shaded region indicates 95% confidence intervals for a linear correlation between $P_{wash\text{-}out}$ vs dP$_{sIONP}$/dt, which is expected for stable loading as the resistance at the end of sIONP loading is equal to the resistance during the beginning of washout. The unshaded white boxed region in the lower left corner in (H) indicates the region of optimal loading conditions.

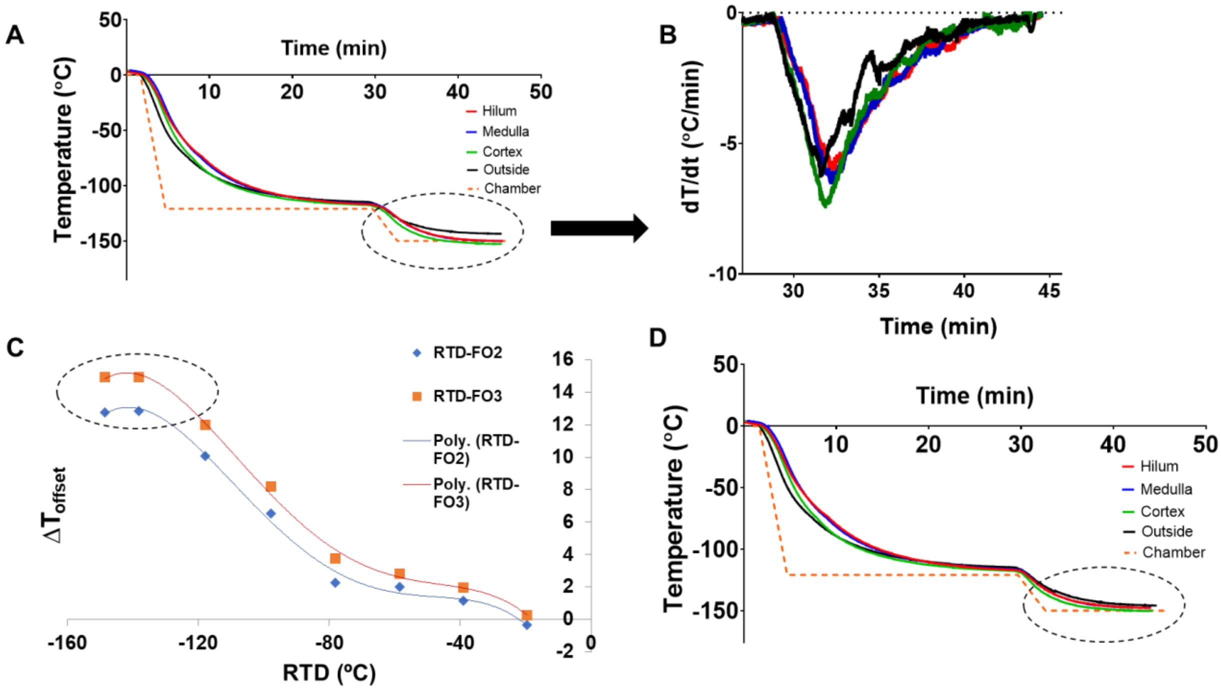

Figure S15. (A) Temperature vs time plot from Figure 3A, reproduced here, showing thermometry at different locations within and outside the kidney during cooling for vitrification. The circled ROI indicates the outside probe at a higher steady-state temperature compared to other probe locations when the kidney is equilibrating to the CRF chamber at -150ºC. (B) Rate of decrease in temperature measured at different probe locations in the temperature range of -121 to

-150ºC (circled region in (A)). The rates indicate that the outside probe is cooling at a similar rate compared to other probe locations. (C) Offset measured in two fiber-optic probes in the -20 to -150ºC temperature range, when calibrated against a Pt100 RTD. The difference in offsets between the two fiber-optic probes used here increases as the temperature decreases. (D) Temperature vs time plot as in (A) after applying probe-specific offset correction.

Table S1. Thermal properties of VS55

Material	Thermal Conductivity: k ($W\ mK^{-1}$)	Specific Heat: C_p ($J\ kg^{-1}\ K^{-1}$)	Density: ρ ($kg\ m^{-3}$)
**Kidney[12,47,49] (assumed equilibrated with VS55)**	0.3	3.011(-1.5ºC)	1100
**Surrounding Solution VS55[47] (Bag)**		2.925(-21ºC)<br>2.819(-44.5ºC)<br>2.715(-78.8ºC)<br>2.968(-118ºC)<br>1.134(-128ºC)<br>0.985(-150ºC)	

HMP CPA Processing Step	Time (min)
**Loading**	
18.75% VS55	15
25% VS55	15
50% VS55	15
75% VS55	15
100% VS55 (8.4 M)	15
**Washout**	

Table S2. VS55 Loading and wash-out steps during hypothermic perfusion

75% VS55	15
50% VS55	15
25% VS55	15
EC	15-30
**Total**	135-150

CPA	Components	Carrier buffer	Total conc.	CCR (°C min^{-1})	CWR (°C min^{-1})
VS55	Dimethyl sulfoxide (3.1 M)<br>Formamide (3.1 M)<br>Propylene Glycol (2.21 M)	Euro-Collins (EC)	8.4 M	-2.5	50

Table S3. VS55 composition and properties

Variable	Range or Specification
Perfusate temperature	0-4°C
Perfusate	VS55 (tables S2 & S3)
Perfusion loading	Step-loading VS55 (table S2)
CPA flow rate	1.5 ml min^{-1} (constant)
P.V. pressure (mm Hg)	0-100 mm Hg (variable with steps)

Table S4. Hypothermic kidney perfusion parameters

Cryopreservation Step	Cold Ischemic Time (mins)
Cold storage → Perfusion	≤ 20
Perfusion → onset of ramp cooling in CRF	17.5 ± 2.5

Table S5. Ischemic times during cryopreservation steps

	CRF Cooling	LN2 Plunge	Nanowarming	Conv. Warming
**Governing Eq.**	$q_v''' = 0$	$q_v''' = 0$	$q_v''' = SAR_{Fe}.C_i$	$q_v''' = 0$
**Boundary Condition**	$-k\frac{\partial T}{\partial r} = h_{eff}\,(T - T_{CRF})$<br>$h_{eff} = 150\;W\;m^{-2}K^{-1}$<br>$T_{CRF}$ *from experiment*	$\mathrm{T_s} = -196°\mathrm{C}$	$-k\frac{\partial T}{\partial r} = h_{eff}\,(T - T_\infty)$<br>$h_{eff} = 15\;W\;m^{-2}K^{-1}$<br>$T_\infty = 20°C$	$-k\frac{\partial T}{\partial r} = h_{eff}\,(T - T_\infty)$<br>$h_{eff} = 5000\;W\;m^{-2}K^{-1}$<br>$T_\infty = 37°C$
**Initial Condition**	$\mathrm{T} = 4°\mathrm{C}$	$\mathrm{T} = 4°\mathrm{C}$	$\mathrm{T} = -138°\mathrm{C}$	$\mathrm{T} = -138°\mathrm{C}$

Table S6. Governing equations for thermal modeling during cooling and rewarming

Table S7. Statistical tests and p-values for significant differences

Figure	p-value	Test	Groups compared
4B	<0.0001	ANOVA with Tukey	Convective vs Outside
	0.0004	ANOVA with Tukey	Cortex vs Outside
	0.0001	ANOVA with Tukey	Medulla vs Outside
	0.0008	ANOVA with Tukey	Hilum vs Outside
4C	0.0009	Unpaired t test	Convective vs Nanowarmed
S3	0.0231	ANOVA with Tukey	Control vs Washout
	0.0002	ANOVA with Tukey	Control vs Blockage
	0.0035	ANOVA with Tukey	Washout vs Blockage
S11	0.0039	ANOVA with Tukey	Control vs Nanowarmed (no washout)
	0.0002	ANOVA with Tukey	Control vs Nanowarmed (washout)
	<0.0001	ANOVA with Tukey	Control vs Convectional warmed
	0.0039	ANOVA with Tukey	CPA Load-Washout vs Nanowarmed (washout)

	<0.0001	ANOVA with Tukey	CPA Load-Washout vs Convectional warmed
	<0.0001	ANOVA with Tukey	Nanowarmed (no washout) vs convectional warmed
	0.0004	ANOVA with Tukey	Nanowarmed (washout) vs convectional warmed
